# Invasion dynamics of super invaders: elimination of Allee effects by a strategy at the range boundary

**DOI:** 10.1007/s00285-025-02269-y

**Published:** 2025-10-28

**Authors:** Yihong Du, Ling Li, Wenjie Ni, Narges Shabgard

**Affiliations:** 1 School of Science and Technology, University of New England, Armidale, NSW 2351 Australia; 2 College of Science, Nanjing Agricultural University, Nanjing, China

**Keywords:** Invasion dynamics, Allee effects, Reaction–diffusion equation, Free boundary, 35B40, 35K55, 35R35

## Abstract

We consider a reaction–diffusion model with free boundaries in one space dimension for a single population species with density *u*(*t*, *x*) and population range [*g*(*t*), *h*(*t*)]. The equations governing the evolution of the range boundary are deduced from the biological assumption that the species maintains its population density at a fixed positive level at the range boundary by advancing or retreating the fronts. Our mathematical results suggest that the Allee effects are eliminated if the species maintains its population density at suitable levels at the range boundary, namely with such a strategy at the range edge the species can invade the environment successfully with all admissible initial populations, exhibiting the dynamics of super invaders. Numerical simulations are used to help understand what happens if the population density levels at the range boundary are maintained in other ranges.

## Introduction

Understanding the dynamical behaviour of invasive species is a central problem in invasion biology, and reaction–diffusion equations have proven to be a useful tool for this purpose (Shigesada and Kawasaki 1997; Cantrell and Cosner 2003; Lewis et al. 2016). In the existing reaction–diffusion models for propagation, the long-time dynamics is primarily determined by the reaction terms (or growth terms in the context of population dynamics), and three types of reaction terms have been widely used, namely monostable, bistable and combustion types of reactions. The last two types of reaction terms can capture the so called Allee effects in population dynamics, which assert that small population size/density may cause inbreeding depression leading to negative growth and eventual vanishing of a species.

However, our analysis of a reaction–diffusion model with free boundaries in this paper suggests that an invasive species may eliminate the Allee effects and spread successfully by a strategy at its range boundary alone. More precisely, Allee effects can be avoided if members of the species at the edge of the population range maintain a favourable density there by advancing or retreating the fronts, and this strategy at the range edge will lead to consistent successful invasion. This appears to be a new phenomenon in reaction diffusion models, and we hope it may shed some light on the understanding of the mechanisms of biological invasion.

Indeed, the assumption here means that for members of the species near the range boundary, their movement in space is governed by two factors: (a) random dispersal similar to all other members, and (b) keeping the density at the front at a preferred level. These two factors are balanced at the range boundary by the species moving the front forward or backward, which is the main reason for the super invasion dynamics.

To put this research into perspective, let us briefly recall some background for the modelling of species spreading.

### Fisher-KPP type reaction–diffusion models

Starting from the pioneering works of Fisher (1937) and Kolmogorov et al. (1937), the spreading behaviour of an invading or new species has been widely modelled by the Cauchy problem

1.1 $$\begin{aligned} \left\{ \begin{array}{ll} U_t=d\Delta U+f(U) & \text{ for } x\in {\mathbb {R}},\; t>0,\\ U(0,x)=U_0(x) & \text{ for } x\in {\mathbb {R}}, \end{array} \right. \end{aligned}$$

where *U*(*t*, *x*) stands for the population density of the concerned species at time *t* and spatial location *x*, with initial population density $U_0(x)$ assumed to be nonnegative with nonempty compact support, to represent the fact that initially the population exists only locally in space. Fisher (1937) assumed $f(U)=U(1-U)$ and KPP (Kolmogorov et al. 1937) allowed more general functions *f* but with similar behaviour; they all belong to the more general class of monostable functions, which are functions with the following properties:

$$\begin{aligned} \mathbf{(f_m):} \ \ \ f\in C^1,\ f(0)=f(1)=0,\ f'(0)>0>f'(1),\; (1-u)f(u)>0 \text{ for } u\in (0,1)\cup (1,\infty ). \end{aligned}$$

A striking feature of (1.1) with *f* satisfying $\mathbf{(f_m)}$ is that it predicts consistent successful spreading with an asymptotic spreading speed: There exists $c^*>0$ such that for any small $\epsilon >0$,

1.2 $$\begin{aligned} \lim _{t\rightarrow \infty ,|x|<(c^*-\epsilon )t}U(t,x)=1,~~~ \lim _{t\rightarrow \infty ,|x|\ge (c^*+\epsilon )t}U(t,x)=0. \end{aligned}$$

This fact was proved by Aronson and Weinberger (1975), Aronson and Weinberger (1978), where $c^*$ was first determined independently by Fisher (1937) and KPP (Kolmogorov et al. 1937) in 1937, who found in Fisher (1937), Kolmogorov et al. (1937) that (1.1) admits a traveling wave $U(t,x)=\phi (x-ct)$ with $\phi (-\infty )=1$ and $\phi (\infty )=0$ if and only if $c\ge c^*:=2\sqrt{f'(0)d}$ and they further claimed that $c^*$ is the spreading speed of the species. The existence of a spreading speed was supported by numerous observations of real world examples, the most well-known being the spreading of muskrats in Europe in the early 20th century; see Skellam (1951), Shigesada and Kawasaki (1997) for more details.

The above result of Aronson and Weinberger on (1.1) has subsequently been proved to be rather robust; for example, the existence of an asymptotic spreading speed has been established for propagation in various heterogeneous environments (see, e.g., Berestycki et al. 2005a, b; Liang and Zhao 2007; Weinberger 1982, 2002).

### Allee effects

The growth function *f* in (1.1) being monostable means, in biological terms, that the environment is favourable for the growth of the species as long as its density is between 0 and 1, with 1 standing for the normalised carrying capacity of the environment. However, it has been observed that very often small population density may cause inbreeding depression which leads to negative growth or vanishing of a species; such a phenomenon is known as the Allee effects in the literature (Allee and Bowen 1932; Kramer et al. 2018). These effects can be captured by (1.1) when *f* is of bistable type, namely it has the following properties:

$$\begin{aligned} (\mathbf {f_b})\ \ \left\{ \begin{aligned}&f\in C^1,\ f(0)=f(\theta )=f(1)=0\ \text {for some}\ \theta \in (0,1), \ \quad f'(0)<0,\quad f'(1)<0,\\&f(u)< 0\ \text {in} \ (0,\theta )\cup (1,\infty ), \quad f(u)>0\ \text {in} \ (\theta ,1), \quad \int _{0}^{1} f(s) ds >0. \end{aligned} \right. \end{aligned}$$

The constant $\theta$ here is known as the Allee threshold density, below which the population has negative growth. It was shown in Aronson and Weinberger (1975), Aronson and Weinberger (1978) that when *f* satisfies $\mathbf{(f_b)}$, for small initial population $U_0(x)$ the species vanishes eventually ($U(t,x)\rightarrow 0$ as $t\rightarrow \infty$), and the population spreads successfully $(U(t,x)\rightarrow 1$ as $t\rightarrow \infty$) when $U_0(x)$ is large. The sharp threshold results in Du and Matano (2010) further imply that generically only these two types of long-time dynamical behaviour are possible. Moreover, as in the monostable case, when spreading is successful, there is also a spreading speed determined by the associated traveling wave problem (Aronson and Weinberger 1975, 1978), and the density function *U*(*t*, *x*) for large *t* is well approximated by the wave profile function (Fife and McLeod 1977).

The stationary problem of (1.1) with a bistable *f* also arises in material science and known as the Allen-Cahn equation (Allen and Cahn 1979; Fonseca and Tartar 1989). In combustion theory, (1.1) is used to model the temperature change with time, and the nonlinear function *f* in such a context is usually assumed to be of combustion type (Kanel’ 1964), characterised by the following properties:

$$\begin{aligned} (\mathbf {f_c})\ \ \left\{ \begin{aligned}&f\in C^1, \ f(u)\equiv 0\ \text {in} \ [0,\theta ]\ \text { for some}\ \theta \in (0,1), \ f(1)=0> f'(1),\\&f(u)>0\ \text {in} \ (\theta ,1), \quad f(u)<0\ \text {in} \ (1,\infty ). \end{aligned} \right. \end{aligned}$$

In this context, $\theta$ is known as the ignition temperature. Qualitatively, the long-time dynamics of (1.1) with *f* of $(\mathbf{f_c})$ type is similar to the situation that *f* is of bistable type $(\mathbf{f_b})$; namely for small initial function $U_0(x)$, vanishing happens ($U(t,x)\rightarrow 0$ as $t\rightarrow \infty$), and the spreading is successful $(U(t,x)\rightarrow 1$ as $t\rightarrow \infty$) when $U_0(x)$ is large (see, e.g., Kanel’ 1964; Aronson and Weinberger 1975, 1978), and the sharp threshold results in Du and Matano (2010) imply that generically only these two types of long-time dynamical behaviour are possible.

In biological terms, a combustion type of *f* can be viewed as reflecting some kind of weak Allee effects, while a bistable *f* reflecting strong Allee effects.

In this paper, we propose to use the following more general class of functions to reflect Allee effects:

$$\begin{aligned} (\mathbf{f_{A}}):\ \ \left\{ \begin{aligned}&f\in C^1,\ \ f(0)=f(\theta )=f(1)=0\ \text {for some}\ \theta \in [0,1), \quad f'(0)\le 0,\quad f'(1)<0,\\&f(u)\le 0\ \text {in} \ [0,\theta ], \quad f(u)>0\ \text {in} \ (\theta ,1), \quad f(u)<0\ \text {in} \ (1,\infty ),\ \int _0^1f(s)ds>0. \end{aligned} \right. \end{aligned}$$

We will henceforth call functions satisfying $\mathbf{(f_{A})}$ of Allee type.

For each *f* of type $\mathbf{(f_A)}$, since $\displaystyle \int _0^u f(s)ds\le 0$ for $u\in [0, \theta ]$ and $\displaystyle \int _0^1f(s)ds>0$, it is easily seen that there exists a unique $\theta ^*=\theta ^*_f\in [\theta , 1)$ such that

1.3 $$\begin{aligned} \int _0^{\theta ^*}f(s)ds=0,\ \int _0^uf(s)ds>0 \text{ for } u\in (\theta ^*, 1]. \end{aligned}$$

Clearly $\mathbf{(f_{A})}$ functions include those in $\mathbf{(f_{b})}$, and as mentioned above, we call this type of growth functions strong Allee type. If *f* satisfies $\mathbf{(f_{A})}$ but not $\mathbf{(f_{b})}$, we will henceforth say it is of weak Allee type, which includes in particular functions satisfying $\mathbf{(f_{c})}$ or satisfying $\mathbf{(f_{A})}$ with $\theta =0$ (which implies $f'(0)=0$); for these two special classes of weak Allee functions clearly $\theta ^*=\theta$. In general, from (1.3) we have

$$\begin{aligned} \theta ^*_f\in {\left\{ \begin{array}{ll} [\theta , 1) & \text{ if } f \text{ is } \text{ of } \text{ weak } \text{ Allee } \text{ type },\\ (\theta , 1) & \text{ if } f \text{ is } \text{ of } \text{ strong } \text{ Allee } \text{ type. } \end{array}\right. } \end{aligned}$$

### Reaction–diffusion models with a Stefan type free boundary

To describe spreading in population dynamics, the models in the previous subsections have a shortcoming: They do not give the precise location of the evolving population range, since although $U(0,x)=U_0(x)$ has compact support, for any $t>0$, one has $U(t,x)>0$ for all $x\in {\mathbb {R}}$. Therefore the population range determined by (1.1) is $\Omega (t):=\{x: U(t,x)>0\}={\mathbb {R}}$ once $t>0$, although $\Omega (0)$ is a bounded set by assumption ($U_0(x)$ has compact support). To avoid this shortcoming, one may nominate a small constant $\sigma \in (0,1)$, and regard

$$\begin{aligned} \Omega _\sigma (t):=\{x: U(t,x)>\sigma \} \end{aligned}$$

as the population range, which is a bounded set for all $t>0$. Then

$$\begin{aligned} \Gamma _\sigma (t):=\{x: U(t,x)=\sigma \} \end{aligned}$$

can be viewed as the spreading front, and (1.2) implies that for any small $\epsilon >0$ and all large $t>0$,

$$\begin{aligned} \Gamma _\sigma (t)\subset \{x: (c^*-\epsilon )t\le |x|\le (c^*+\epsilon )t\}. \end{aligned}$$

Therefore (1.2) can be interpreted as saying that the fronts go to infinity with asymptotic speed $c^*$. It should be noted that $c^*$ is determined by the associated traveling wave problem, and is independent of the choice of $\sigma \in (0,1)$ in $\Omega _\sigma$. Interestingly, however, in the real world spreading speed is obtained from observations of the actual range expansion; see Sect. 1.4 below for more details.

To avoid using an artificial number $\sigma$ in the expression of the population range, Du and Lin (2010) modified (1.1) into a free boundary problem of the form

1.4 $$\begin{aligned} {\left\{ \begin{array}{ll} u_t-du_{xx}=f(u), & t>0, \ g(t)<x<h(t),\\ u(t,g(t))= u(t,h(t))=0,& t>0,\\ g'(t)=-\mu u_x(t,g(t)), & t>0,\\ h'(t)=-\mu u_x(t,h(t)),& t>0,\\ u(0,x)=u_0(x),\ -g(0)=h(0)=h_0,& -h_0\le x\le h_0, \end{array}\right. } \end{aligned}$$

where $\mu >0$ is a constant and the population range is explicitly given by the interval [*g*(*t*), *h*(*t*)] in the model. The free boundary conditions in (1.4) coincide with the Stefan conditions in the classical free boundary model for melting of ice in contact with water, and for the biological setting here, they can be derived from some biological assumptions (Bunting et al. 2012): If we assume that in order to expand the population range, the species sacrifices *k* units of its population at the range boundary per unit of time and space, then $\mu =d/k$.

For *f* of monostable type, it has been shown (see Du and Lou 2015) that (1.4) exhibits a spreading-vanishing dichotomy for its long-time dynamics: As $t\rightarrow \infty$, either [*g*(*t*), *h*(*t*)] converges to a finite interval $[g_\infty , h_\infty ]$ and $u(t,x)\rightarrow 0$ uniformly (the vanishing case), or $[g(t), h(t)] \rightarrow {\mathbb {R}}$ and $u(t,x)\rightarrow 1$ (the spreading case). Moreover, in the latter case, there exists an asymptotic spreading speed determined by an associated traveling wave problem (called a semi-wave problem in the literature partly due to the fact that the wave profile function is only defined over the half line, partly due to the need to distinguish it from the classical traveling wave problem of Fisher and KPP). Furthermore, it was shown in Du et al. (2014) that when spreading occurs, the density function *u*(*t*, *x*) is well approximated by the semi-wave profile function as $t\rightarrow \infty$.

When *f* is of the type $\mathbf{(f_b)}$ or $\mathbf{(f_c)}$, it follows from Du and Lou (2015) that for all small initial population $u_0(x)$ vanishing happens, and for all large $u_0(x)$ spreading happens; the sharp transition result in Du and Lou (2015) implies that generically these are the only possible long-time behaviour of (1.4). The results in Du et al. (2014) on the spreading speed and population density also apply to these cases when spreading is successful.

### Super invaders

The dynamics displayed by the models in the previous two subsections, especially those with *f* of bistable type $\mathbf{(f_b)}$ or of combustion type $\mathbf{(f_c)}$, where the population vanishes when its initial size is small and spreads successfully when the initial size is large, agrees with many real world observations (Duncan et al. 2003); for example, 744 of 1466 (51%) introduction events of birds in the global data set analyzed by Blackburn and Duncan (2001) did not result in establishment.

**Fig. 1:**
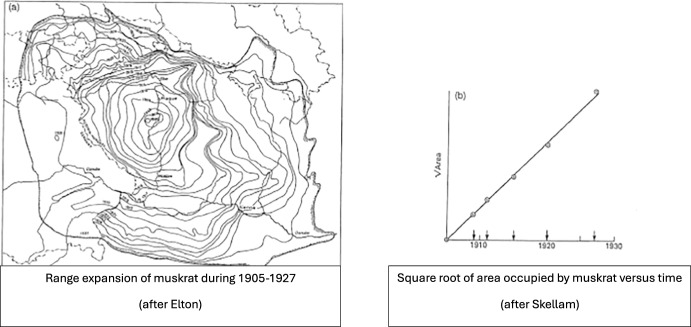
Evolution of the population range of muskrats in Europe during 1905–1927

However, there are examples of super invaders^1^ which spread successfully against all odds. The spreading of muskrats in Europe in the early 1900 s is an example with very small initial size: In 1905, five escaped muskrats from a farm near Prague resulted in a successful invasion of the entire European continent within less than 3 decades (Shigesada and Kawasaki 1997; Lewis et al. 2016). This example enabled (Skellam 1951) to observe the phenomenon of spreading with an asymptotic speed (as claimed by Fisher and KPP in 1937): He calculated the area of the muskrat range from a map obtained from field data, took the square root (which gives a constant multiple of the range radius) and plotted it against years, and found that the data points lay on a straight line; see Fig. 1.

**Fig. 2:**
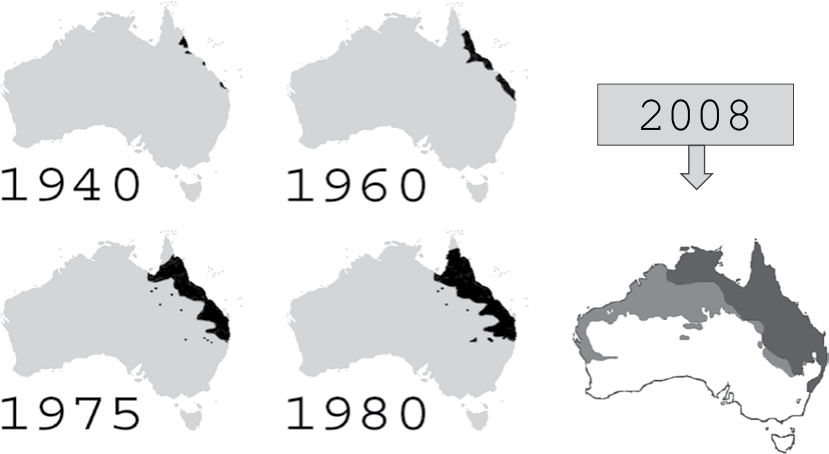
Evolution of the population range of cane toads over the years (graphs taken from the internet)

Another well known super invader is the cane toads in Australia. Introduced in Australia from Hawaii in 1935, as an attempt to control “cane beetles" in sugar cane fields of Northern Queensland, the cane toads ended up a pest causing significant environmental detriment (and with no evidence that they have affected the number of cane beetles which they were introduced to prey upon). The invasion of cane toads in Australia is continuing, at an estimated speed of 40–50 kms per year; see Fig. 2.

Based on field observations, biologists have noticed several distinctive behaviours of the invading cane toads, which include:

- toads at the front have longer legs and move faster (Phillips et al. 2006),
- toads can invade into much less favourable territory with lower speed, including temporary front retreats (Macgregor et al. 2021).

### A free boundary model for super invaders

In Du (2024), the following variation of (1.4) was considered,

1.5 $$\begin{aligned} {\left\{ \begin{array}{ll} u_t-du_{xx}=f(u), & t>0,\; g(t)<x<h(t),\\ u(t,g(t))=u(t,h(t))=\delta , & t>0,\\ g'(t)=-\frac{d}{\delta }u_{x}(t,g(t)), & t>0,\\ h'(t)=-\frac{d}{\delta }u_x(t,h(t)), & t>0,\\ -g(0)=h(0)=h_0, u(0,x)=u_0(x), & -h_0\le x\le h_0. \end{array}\right. } \end{aligned}$$

Similar to (1.4), here *u*(*t*, *x*) stands for the population density and [*g*(*t*), *h*(*t*)] represents the population range. The initial function $u_0$ is assumed to belong to

$$\begin{aligned} {X}(h_0):=\{\phi \in C^2([-h_0, h_0]): \phi (\pm h_0)=\delta , \ \phi >0 \ \text {in} \ [-h_0, h_0]\}. \end{aligned}$$

While the population range in (1.4) always expands as time increases, this is no longer the case for (1.5), where the fronts $x=h(t)$ and $x=g(t)$ may advance or retreat as time increases, which is a main new feature of the current model. The constant $\delta \in (0,1)$ in (1.5) represents the species’ preferred density, and the equations governing the evolution of the free boundaries $x=g(t)$ and $x=h(t)$, namely the second, third and fourth equations in (1.5), can be deduced from the biological assumption that the species maintains its preferred density $\delta$ at the range boundary by advancing or retreating the fronts; see Du (2024) for a detailed deduction and more background. This assumption means that for members of the species near the range boundary, their movement in space is governed by two factors: (a) random movement similar to all other members, and (b) advance or retreat to keep the density at the front at the preferred level $\delta$.

When the growth function satisfies $\mathbf{(f_m)}$, it was shown in Du (2024) that the unique solution (*u*(*t*, *x*), *g*(*t*), *h*(*t*)) of (1.5) exhibits successful spreading for all admissible initial data (namely for every $u_0\in X(h_0)$ with $h_0>0$). We will show in this paper that successful spreading also happens consistently when *f* satisfies $\mathbf{(f_A)}$ and $\delta \in (\theta ^*_f, 1)$, and so (1.5) is capable of exhibiting the dynamics of super invaders.

### Main results and structure of the paper

The main purpose of this paper is to demonstrate that successful spreading is always achieved by (1.5) when *f* is of Allee type, namely $\mathbf{(f_{A})}$ holds, provided that the preferred density $\delta$ at the range boundary satisfies $\delta \in (\theta ^*_f, 1)$. In other words, the Allee effects exhibited in (1.1) and (1.4) can be eliminated if the species chooses the strategy to advance or retreat the boundary of the population range to keep the density there at a level $\delta \in (\theta ^*_f, 1)$.

#### Theorem 1.1

Suppose that *f* is of Allee type $\mathbf{(f_A)}$ and $\delta \in (\theta _f^*, 1)$. Then for every initial function $u_0 \in X(h_0)$, (1.5) has a unique solution $(u(t,x), g(t), h(t))$ defined for all $t>0$. Moreover, as $t \rightarrow \infty$,

$$\begin{aligned} (g(t), h(t)) \rightarrow (-\infty , \infty ), \quad u(t, x) \rightarrow 1 \quad \text {locally\ uniformly\ in }\ x \in {\mathbb {R}}. \end{aligned}$$

Furthermore, there exist some constants ${\hat{h}}, {\hat{g}} \in {\mathbb {R}}$ such that

$$\begin{aligned}&\lim _{t \rightarrow \infty }[h(t) - c^* t] = {\hat{h}}, \quad \lim _{t \rightarrow \infty } h'(t) = c^*,\\&\lim _{t \rightarrow \infty }[g(t) + c^* t] = {\hat{g}}, \quad \lim _{t \rightarrow \infty } g'(t) = -c^*,\\&\lim _{t \rightarrow \infty } \sup _{x \in [0, h(t)]} |u(t, x) - q^*( h(t) - x)| = 0,\\&\lim _{t \rightarrow \infty } \sup _{x \in [g(t), 0]} |u(t, x) - q^*( x - g(t))| = 0, \end{aligned}$$

where $(c^*, q^*)$ is the unique solution pair $(c,q)$ of

1.6 $$\begin{aligned} {\left\{ \begin{array}{ll} dq'' - cq' + f(q) = 0, \quad q > 0 \quad \text {in }\ (0,\infty ), \\ q(0) = \delta , \quad q(\infty ) = 1, \quad q'(0) = \frac{c \delta }{d},\quad q'>0\ \text{ in }\ [0,\infty ). \end{array}\right. } \end{aligned}$$

To emphasise the new dynamical feature of (1.5), the part of Theorem 1.1 on the long-time dynamical behaviour of (1.5) is proved in Sect. 2, while the existence and uniqueness result for (1.5) is left to Sect. 3, where a more general system (3.1) will be treated. In Sect. 2, we also discuss, through numerical simulations, what happens to (1.5) if the assumption $\delta \in (\theta _f^*, 1)$ is not satisfied.

Much of the mathematical techniques to treat (1.5) can be extended to study a more general version of (1.5), where heterogenous environment can be included. In Sect. 3, we first consider the existence and uniqueness of a solution to the more general system (3.1) (which implies the corresponding conclusion for (1.5) stated in Theorem 1.1), then we obtain a more general result (than Theorem 1.1) on the elimination of Allee effects for (3.1) (see Theorem 3.3). Moreover, we obtain some further theoretical results on the long-time dynamics of (3.1) (see Theorems 3.4 and 3.5) and confirm several conjectures listed in Sect. 2. These imply, in particular, that in Theorem 1.1, if the assumption on $\delta$ is changed to $\delta >1$ then vanishing always happens, and a transition phenomenon appears when $\delta =1$. The proofs of these theoretical results are postponed to an appendix, consisting of Sect. 5, the last section of the paper. While some of the techniques in Sect. 5 are based on existing ones, several new techniques are introduced in the proof of Lemma 5.5 and in Sects. 5.5 and 5.6, which may find applications in future work.

In Sect. 4, our results are summarised and explained further in biological terms. Possible directions of related future research and comparison to some existing works are also discussed there.

## Longtime dynamics of (1.5)

### Proof of Theorem 1.1

In order not to interrupt thoughts with too many technicalities, and also to make the reading more accessible to biologically oriented readers, the existence and uniqueness of the solution to (1.5) is proved in Sect. 3, where a more general problem than (1.5) will be considered.

Here we only prove the longtime behavior of the solution. The strategy is to reduce the problem to the case of (1.5) with a monostable growth function, and then all the conclusions will follow from Du (2024).

A key step is to show that when $\mathbf{(f_A)}$ holds and $\delta \in (\theta ^*_f, 1)$, there exists $T_0>0$ such that

2.1 $$\begin{aligned} u(t, x) \ge \delta \text{ for } t\ge T_0 \text{ and } x\in [g(t), h(t)]. \end{aligned}$$

Once (2.1) is established, the conclusions will follow from the monostable case in Du (2024) as explained below. Let

$$\begin{aligned} ({\tilde{u}}(t,x), {\tilde{g}}(t),{\tilde{h}}(t)):=(u(T_0+t, x), g(T_0+t), h(T_0+t)). \end{aligned}$$

Then clearly

2.2 $$\begin{aligned} {\left\{ \begin{array}{ll} {\tilde{u}}_t-d {\tilde{u}}_{xx}=f({\tilde{u}}), & t>0,\; {\tilde{g}}(t)<x< {\tilde{h}}(t),\\ {\tilde{u}}(t, {\tilde{g}}(t))={\tilde{u}}(t,{\tilde{h}}(t))=\delta , & t>0,\\ {\tilde{g}}'(t)=-\frac{d}{\delta } {\tilde{u}}_{x}(t, {\tilde{g}}(t)),& t>0,\\ {\tilde{h}}'(t)=-\frac{d}{\delta }{\tilde{u}}_x(t, {\tilde{h}}(t)), & t>0,\\ {\tilde{g}}(0)=g(T_0),\ {\tilde{h}}(0)=h(T_0), \ {\tilde{u}}(0,x)=u(T_0, x), & g(T_0)\le x\le h(T_0). \end{array}\right. } \end{aligned}$$

We now redefine *f*(*s*) for $s\in (0, \delta )$ to obtain a new ${\tilde{f}}(s)$ such that ${\tilde{f}}$ satisfies $\mathbf{(f_m)}$ and ${\tilde{f}}(s)=f(s)$ for $s\ge \delta$. Since ${\tilde{u}}\ge \delta$ by (2.1), we see that $({\tilde{u}}(t,x), {\tilde{g}}(t),{\tilde{h}}(t))$ satisfies (2.2) with *f* replaced by ${\tilde{f}}$. Therefore we can use the results in Du (2024) to conclude that $({\tilde{u}}(t,x), {\tilde{g}}(t),{\tilde{h}}(t))$ has all the properties stated in Theorems 1.2 and 1.4 there, which is equivalent to saying that (*u*, *g*, *h*) has all the properties stated in Theorem 1.1. Let us note that the requirement $h(0)=-g(0)$ in (1.5) (and in Du (2024)) is for convenience only; we can easily recover ${\tilde{h}}(0)=-{\tilde{g}}(0)$ in (2.2) by a suitable translation of the variable *x*.

It remains to prove (2.1). We will need a well known result (see, e.g., Aronson and Weinberger 1978, Theorem 4.1) on traveling wave solutions of

2.3 $$\begin{aligned} u_t - d u_{xx} = F(u), \quad t > 0, \; x \in {\mathbb {R}}, \end{aligned}$$

with $F \in C^1([0, \infty ))$ satisfying the following (unnormalised) bistable condition:

$$\begin{aligned} (\mathbf {F_b}):\ \ \ \ \left\{ \begin{aligned}&F(0) = F(P) = F(Q) = 0 \ \text {with } 0<P<Q<\infty , \quad F'(0)<0,\ F'(Q)< 0, \\&F < 0 \ \text {in } (0, P)\cup (Q,\infty ), \quad F> 0 \ \text {in } (P, Q), \ \int _{0}^{Q} F(s) \, ds > 0. \end{aligned} \right. \end{aligned}$$

#### Lemma 2.1

(Aronson and Weinberger 1978) Let $F$ satisfy $\mathbf {(F_b)}$. Then there exists a constant $c_* > 0$ such that (2.3) has a traveling wave solution $u(t,x)=\phi (x+c_*t)$ with speed $c_*>0$; more precisely, the problem

$$\begin{aligned} {\left\{ \begin{array}{ll} d \phi ''(x) - c_* \phi '(x) + F(\phi (x)) = 0, \quad x \in {\mathbb {R}}, \\ \phi (-\infty ) = 0, \quad \phi (+\infty ) = Q, \end{array}\right. } \end{aligned}$$

admits a solution $\phi \in C^2({\mathbb {R}})$ which is strictly increasing.

We are now ready to prove (2.1). Since $\delta \in (\theta ^*_f, 1)$, we have $\int _0^\delta f(s)ds>\int _0^{\theta _f^*}f(s)ds=0$. Therefore we are able to choose a function ${\hat{f}} \in C^1$ sufficiently close to *f* in $L^1([0,\delta ])$ such that ${\hat{f}}(s) \le f(s)$ for $s \ge 0$, and ${{\hat{f}}}$ satisfies $\mathbf {(F_b)}$ with $(P, Q)=({\hat{\theta }}, \delta )$ for some ${\hat{\theta \in }} [\theta , \delta )\cap (0,\delta )$. In particular, we have ${\hat{f}}(0) = {\hat{f}}({\hat{\theta }}) = {\hat{f}}(\delta )=0$ and ${\hat{f}}(s) > 0$ for $s \in ({\hat{\theta }}, \delta )$. Then, by Lemma 2.1, the following problem

2.4 $$\begin{aligned} \left\{ \begin{aligned}&dq'' - cq' + {\hat{f}}(q) = 0, \quad z \in {\mathbb {R}}, \\&q(-\infty ) = 0, \quad q(\infty ) = \delta , \end{aligned} \right. \end{aligned}$$

has a solution pair $(c,q)=(c_0,q_0)$ with $c_0>0$ and $q_0(\cdot )$ strictly increasing.

Next, we will make use of $q_0(z)$ to construct a lower solution to bound *u*(*t*, *x*) from below. Since $q_0(-\infty ) = 0$, we can choose $L > 0$ sufficiently large such that $q_0(h_0 - L) \le \min _{x \in [-h_0, h_0]} u_0(x)$. Define

$$\begin{aligned} {\underline{u}}(t, x):= \max \{q_0(ct - x - L), q_0(ct + x - L)\}. \end{aligned}$$

Due to ${\hat{f}} \le f$ and ${\underline{u}}_x(t, 0^-) = -q_0'(ct - L)\le 0 \le q_0'(ct - L) = {\underline{u}}_x(t, 0^+)$, it is easy to see that ${\underline{u}}(t, x)$ satisfies (in the weak sense)

$$\begin{aligned} {\underline{u}}_t \le d {\underline{u}}_{xx} + f({\underline{u}}) \quad \text {for } t > 0, \; x \in {\mathbb {R}}. \end{aligned}$$

Additionally, we have

$$\begin{aligned} 0 \le {\underline{u}}(t, x) \le \delta \quad \text {for } x \in {\mathbb {R}},\ \text{ which } \text{ implies } {\underline{u}}(t, x)\le u(t,x) \text{ for } t>0,\ x\in \{g(t), h(t)\},\end{aligned}$$

and

$$\begin{aligned} {\underline{u}}(0, x) = \max \{q_0(-x - L), q_0(x - L)\} \le q_0(h_0 - L) \le u_0(x) \quad \text {for } x \in [-h_0, h_0]. \end{aligned}$$

Therefore we can apply the standard comparison principle over $\{(t, x): t > 0, x \in [g(t), h(t)]\}$ to deduce $u(t, x) \ge {\underline{u}}(t, x)$ in this region.

Moreover, since $q_0(\infty ) = \delta$ and $q_0$ is increasing, we conclude that for large $t > 0$,

$$\begin{aligned} \delta \ge \sup _{x \in {\mathbb {R}}} {\underline{u}}(t, x) \ge \inf _{x \in {\mathbb {R}}} {\underline{u}}(t, x) \ge q_0(ct - L) \rightarrow \delta \quad \text {as } t \rightarrow \infty . \end{aligned}$$

Hence

$$\begin{aligned} \lim _{t \rightarrow \infty } \Vert {\underline{u}}(t,\cdot ) - \delta \Vert _{L^\infty ({\mathbb {R}})} = 0. \end{aligned}$$

Since $\delta > \theta$, there exists $t_0 > 0$ such that

$$\begin{aligned} {u}(t, x) \ge {\underline{u}}(t, x) >\theta \quad \text{ for } t \ge t_0, \; x \in [g(t), h(t)]. \end{aligned}$$

Now, let $m_0:= \min _{x \in [g(t_0), h(t_0)]} u(t_0, x)$. Then $\delta \ge m_0 >\theta$. Consider the auxiliary ODE problem:

$$\begin{aligned} w' = f(w) \text{ for } t > t_0, \qquad w(t_0) = m_0. \end{aligned}$$

Since $f > 0$ in $(\theta , 1)$, the solution *w*(*t*) is increasing in *t*, and $w(t) \rightarrow 1$ as $t \rightarrow \infty$. Thus, there exists $T_\delta \ge t_0$ such that $w(T_\delta ) = \delta$ and $m_0 \le w(t) \le \delta$ for $t \in [t_0, T_\delta ]$. By the comparison principle over the region $\{(t, x): t_0 \le t \le T_\delta , \; g(t) \le x \le h(t)\}$, we obtain $u(t, x) \ge w(t)$ in this region. In particular,

$$\begin{aligned} u(T_\delta , x) \ge w(T_\delta ) = \delta \quad \text{ for } x \in [g(T_\delta ), h(T_\delta )]. \end{aligned}$$

Comparing $u(t, x)$ with the constant function ${\underline{u}}_1(t, x) \equiv \delta$ over $\{(t, x): T_\delta \le t < \infty , \; g(t) \le x \le h(t)\}$, we conclude by the usual comparison principle that $u(t, x) \ge \delta$ for $x \in [g(t), h(t)]$ and $t \in [T_\delta , \infty )$. This proves (2.1), and the proof of Theorem 1.1 is finished except that the existence and uniqueness of the solution to (1.5) will follow from a more general result proved in Sect. 3 below. $\square$

### Numerical simulation and conjectures

One naturally wonders what happens if $\delta \not \in (\theta _f^*, 1)$ in (1.5). Our numerical simulation^2^ on (1.5) with monostable *f*, bistable *f* and combustion *f* suggests the following conjectures.

#### Conjecture 1

If $\delta >1$ and *f* satisfies $\mathbf{(f_m)}$, or $\mathbf{(f_b)}$, or $\mathbf{(f_c)}$, then there exists $\xi ^*\in {\mathbb {R}}$ depending on *f* and $u_0$ such that

$$\begin{aligned} \lim _{t\rightarrow \infty } g(t)=\lim _{t\rightarrow \infty } h(t)=\xi ^*, \ \lim _{t\rightarrow \infty } u(t,x)=\delta\ \text{ uniformly }\ \text{ for }\ x\in [g(t), h(t)]. \end{aligned}$$

Let us note that the above conclusions indicate that the population range vanishes as $t\rightarrow \infty$, and we will called this the **vanishing case** henceforth.^3^ Biologically, this suggests that maintaining a density above the carrying capacity of the environment at the moving range boundary will lead to vanishing of the species.

In our numerical simulations, for monostable *f*, we take

2.5 $$\begin{aligned} f(u)=u(1-u); \end{aligned}$$

for bistable *f*, we take

2.6 $$\begin{aligned} f(u)=u(u-\theta )(1-u) \text{ with } \theta \in (0, 1/2); \end{aligned}$$

and for combustion *f*, we take, with $\theta \in (0,1)$ and $\beta =0.1$,

2.7 $$\begin{aligned} f(u) = {\left\{ \begin{array}{ll} 0 & \text { for } 0\le u \le \theta , \\ \beta (u - \theta )^2 (1 - u) & \text { for } u > \theta . \end{array}\right. } \end{aligned}$$

For the initial function, most of the times we take

2.8 $$\begin{aligned} u_{0}(x)=\delta + \alpha \cos (\frac{\pi x}{2h_{0}})\ \text{ with } \alpha > -\delta ,\ h_0=10. \end{aligned}$$

Since $u_0(x)$ is even, the solution (*u*(*t*, *x*), *g*(*t*), *h*(*t*)) is also even in *x* for any time $t>0$, and so $g(t)=-h(t)$ and $u(t, -x)=u(t,x)$. Therefore we will only consider *u*(*t*, *x*) for $x\ge 0$. This simplifies the simulation and discussions. Note that in such a case, we always have $\xi ^*=0$ in Conjecture 1.

**Fig. 3:**
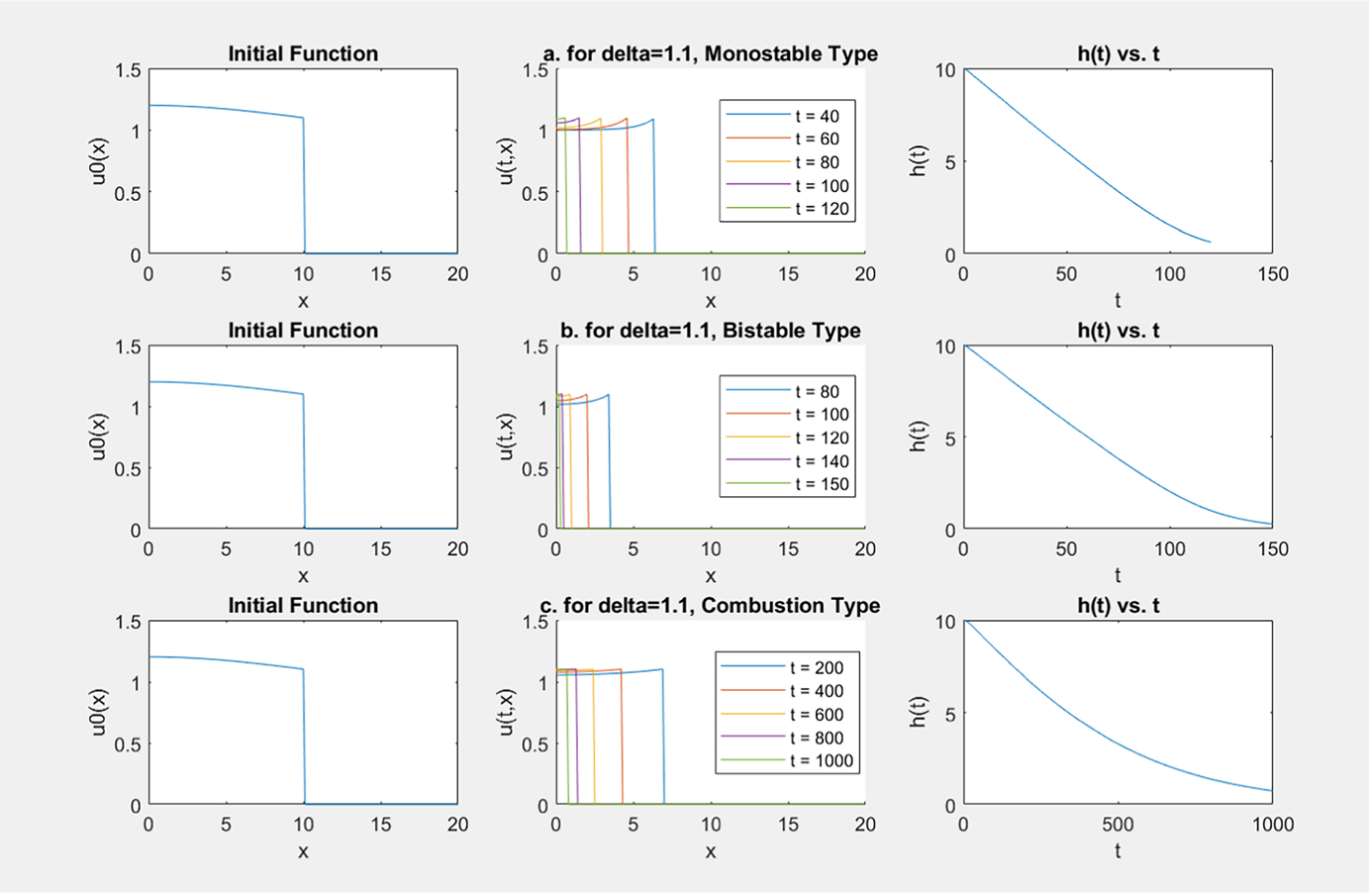
Simulation results with $\delta >1$: vanishing always happens

A sample of our numerical simulations is given in Fig. 3, where the graph of $x\rightarrow u(t,x)$ (for several time moments) and the graph of $t\rightarrow h(t)$ are given. In these simulations, $\theta =0.2$ was taken for bistable and combustion *f* given in (2.6) and (2.7), respectively, and $u_0(x)$ is given by (2.8) with $\alpha =0.1$.

#### Remark 2.2

Conjecture 1 will be confirmed by Theorem 3.4 in Sect. 3, where we also prove that when $\delta =1$, a new transition phenomenon between vanishing and successful spreading happens; see Theorem 3.5.

#### Conjecture 2

When $\mathbf{(f_b)}$ holds and $\delta \in (\theta , \theta _f^*]$, the conclusions in Theorem 1.1 still hold except when $(u_0(x), -h_0, h_0)$ happens to be a stationary solution of (1.5).

This is a technical issue regarding what is the threshold level at the range boundary to eliminate the Allee effect. Theoretically we proved that above $\theta ^*$ is enough, but numerical simulation suggests the lower level $\theta$ is enough. (Conjecture 3 below indicates that below $\theta$ is definitely not enough.) These stationary solutions are exceptional initial values for which successful spreading does not happen, but they are nongeneric and cannot be observed in numerical simulations.

By a phase-plane analysis, it is easy to see that all the stationary solutions of (1.5) with a bistable *f* and $\delta \in (0, 1)$ are found as follows:

(i) If $\delta \in [\theta ^*, 1)$, then there are no stationary solutions.
(ii) If $\delta =\theta$, then the stationary solutions are: $(u, g, h)=(\theta , -h_0, h_0)$, $h_0>0$.
(iii) If $\delta \in (0, \theta ^*)\setminus \{\theta \}$, then the solution *v*(*x*) of the initial value problem
  $$\begin{aligned} dv''+f(v)=0,\; v'(0)=0, v(0)=\delta \end{aligned}$$
  is periodic in *x* with $0<\min v<\theta<\max v<\theta ^*$. Let *L* be the minimal period of *v*; then for every integer $j\ge 1$,
  $$\begin{aligned} (v(x), -jL, jL) \text{ and } \Big (v(x+\frac{L}{2}), -(j-\frac{1}{2})L, (j-\frac{1}{2})L\Big ) \end{aligned}$$
  are stationary solutions of (1.5). There are no other stationary solutions.

**Fig. 4:**
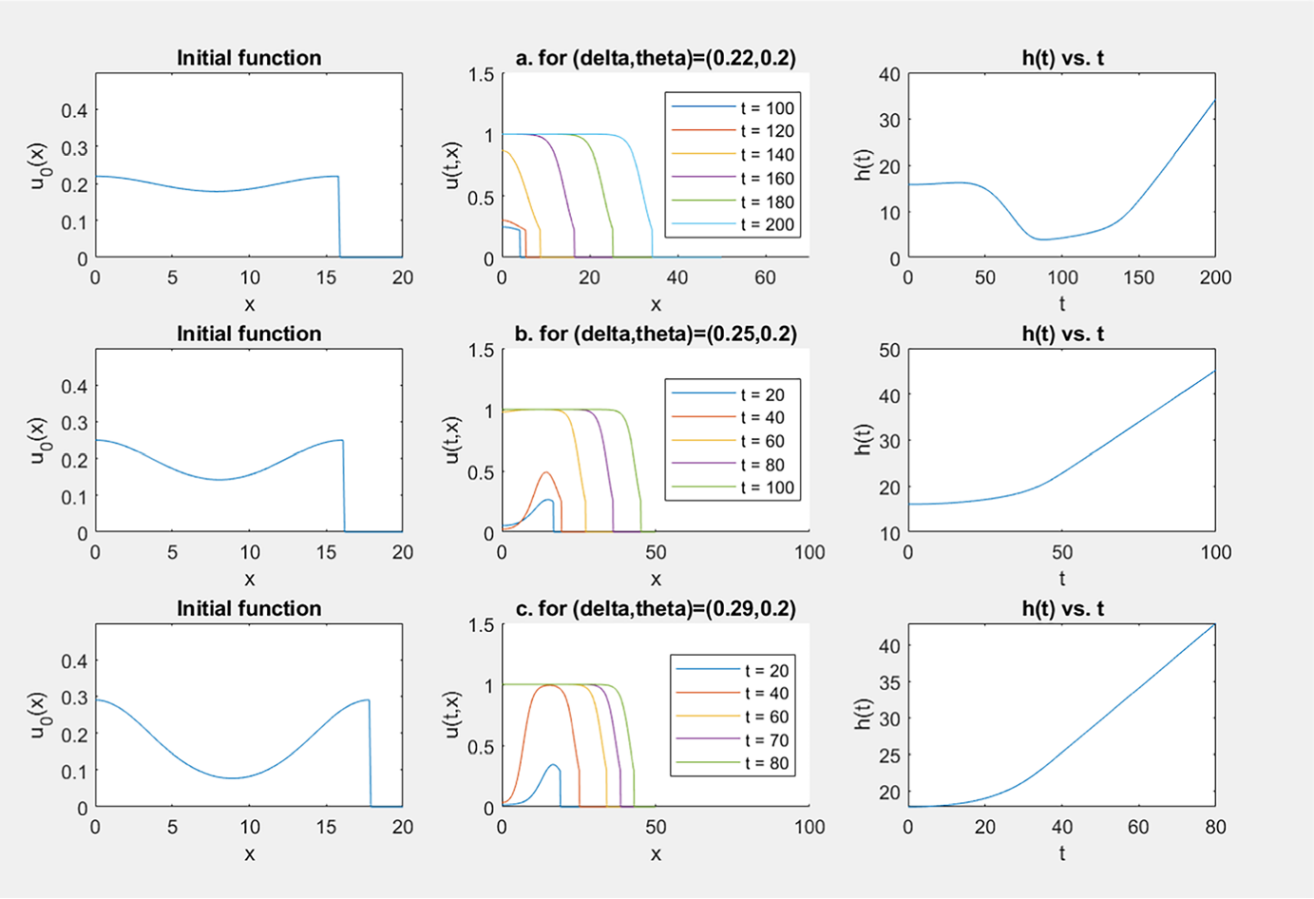
Simulation results for $\delta \in (\theta , \theta ^*)=(0.2, 0.3101)$: spreading always happens

Our numerical simulations for this case always exhibit successful spreading, even when we tried to start from a numerically obtained stationary solution, indicating that the stationary solutions are unstable. A sample of our simulation results for this case are given in Fig. 4, where *f*(*u*) is given by (2.6) and $(u_0(x), -h_0, h_0)$ is given by a numerically obtained stationary solution (for the given value of $\delta$).

#### Conjecture 3

When $\mathbf{(f_b)}$ holds and $\delta \in (0, \theta )$, then, as $t\rightarrow \infty$, vanishing happens if the initial population is small and spreading happens if the initial population is large.

Biologically this suggests that if the density level maintained at the range boundary is not high enough, the Allee effect is not eliminated.

Our simulation results for this case are listed in the table below, where *f*(*u*) and $u_0(x)$ are given by (2.6) and (2.8), respectively.

**Table:** 

$\theta$	$\delta$	$\alpha$	Longtime dynamics
0.2	0.10	0.1935	Spreading
		0.1934	Spreading
		**0.1933**	Vanishing
		0.1932	Vanishing
0.2	0.15	0.1045	Spreading
		0.1044	Spreading
		0.1043	Spreading
		**0.1042**	Vanishing
		0.1041	Vanishing
0.2	0.19	0.0235	Spreading
		0.0234	Spreading
		0.0233	Spreading
		0.0232	Spreading
		0.0231	Spreading
		**0.0230**	Vanishing
		0.0229	Vanishing

**Fig. 5:**
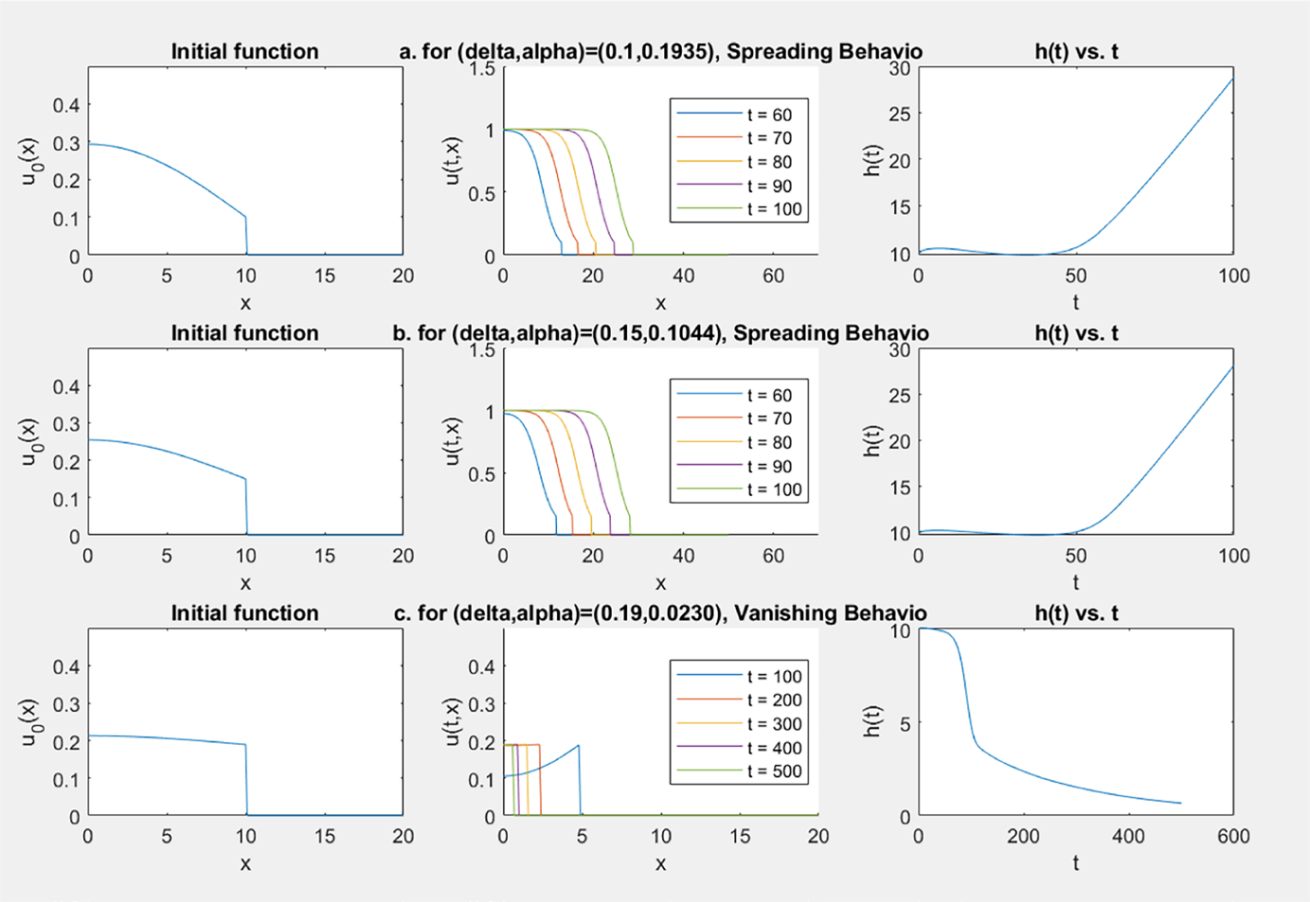
Simulation results for $\delta \in (0,\theta )$ with a bistable *f*(*u*): either spreading or vanishing happens

Some samples of the graphs of $x\rightarrow u(t,x)$ and $t\rightarrow h(t)$ obtained from the simulations listed in this table are given in Fig. 5.

#### Conjecture 4

When $\mathbf{(f_c)}$ holds and $\delta \in (0, \theta _f^*]=(0, \theta ]$, then

(i) $u_0\in X(h_0)$ and $u_0(x)\le \theta$ in $[-h_0, h_0]$ imply that, as $t\rightarrow \infty$, $u(t,x)\rightarrow \delta$ and $[g(t), h(t)]\rightarrow [g_\infty , h_\infty ]$ is a finite interval with
  2.9 $$\begin{aligned} (h_\infty -g_\infty )\delta = \int _{-h_0}^{h_0}u_0(x)dx. \end{aligned}$$
(ii) If $u_0\in X(h_0)$ and $u_0(x)>\theta$ for some $x\in (-h_0, h_0)$, then either spreading happens or the above conclusions in (i) hold except that (2.9) need not be true anymore.

Biologically, this suggests that with a weak Allee response function, if both the initial population density and the density maintained at the range boundary are below the threshold level $\theta$ (case (i)), then the population density over the entire range will stabilise at the boundary level and the population range will also converge to a finite region, but a very unevenly distributed initial population may lead to vanishing (case(ii)).

Our simulation results for this case are listed in the table below, where *f*(*u*) and $u_0(x)$ are given by, respectively, (2.7) and (2.8).

**Table:** 

$\theta$	$\delta$	$\alpha$	Longtime dynamics
0.3	0.28	5.315	Spreading
		5.314	Spreading
		5.313	Spreading
		5.312	Spreading
		**5.311**	Converging to $\delta$
		5.310	Converging to $\delta$
0.3	0.29	4.500	Spreading
		4.400	Spreading
		4.300	Spreading
		**4.299**	Converging to $\delta$
		4.298	Converging to $\delta$

**Fig. 6:**
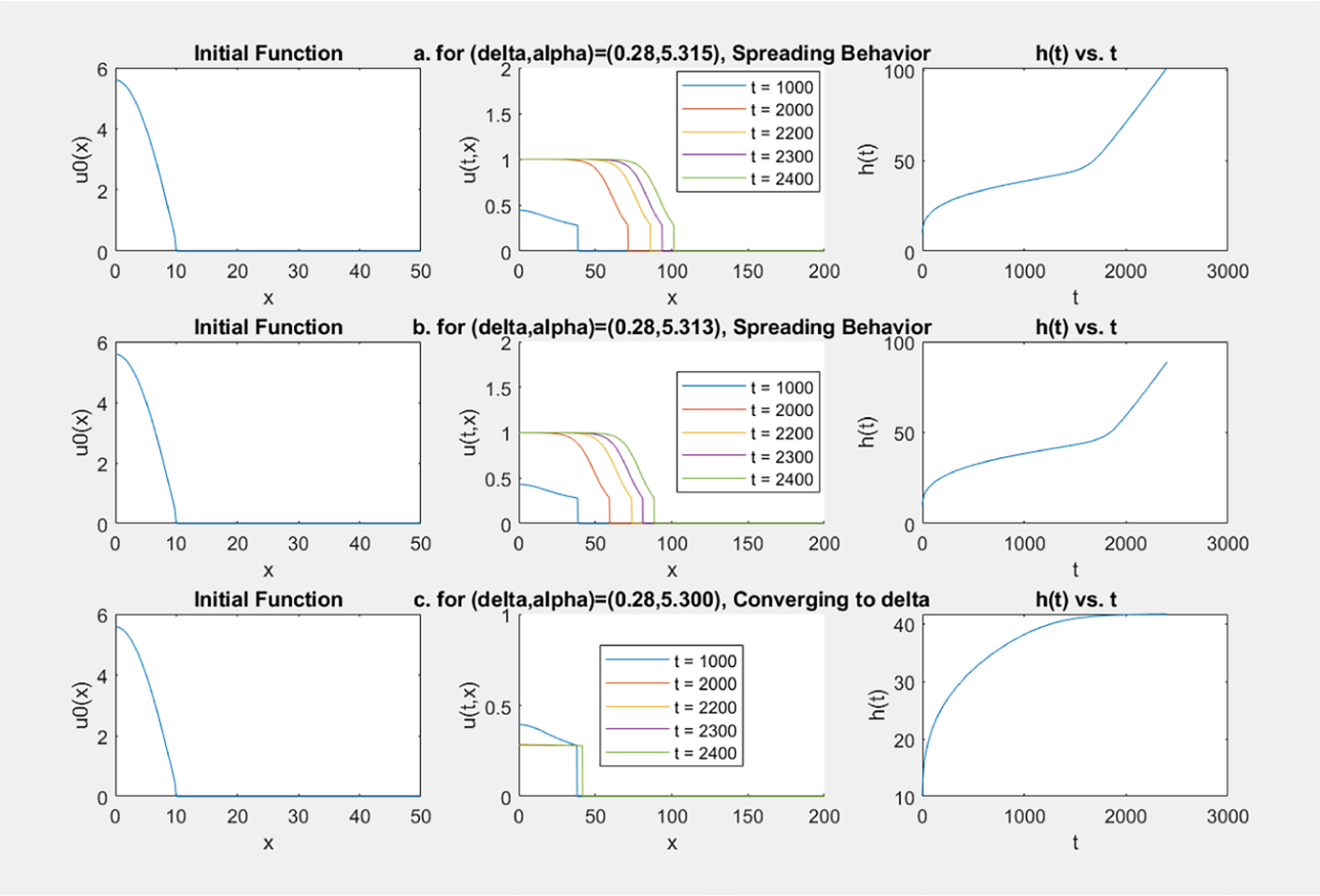
Simulation results for $\delta \in (0,\theta )$ with a combustion *f*(*u*): either spreading or convergence to $\delta$ happens

Some samples of the graphs of $x\rightarrow u(t,x)$ and $t\rightarrow h(t)$ obtained from the simulations listed in this table are given in Fig. 6.

#### Remark 2.3

Part (i) of Conjecture 4 will be rigorously proved in Sect. 3.6.

## Extensions to heterogeneous environment and further theoretical results

In this section, we extend the theoretical results in the previous section to situations involving heterogeneous environment, and obtain several new results including in particular positive answers to Conjecture 1 and Conjecture 4 (i).

We consider the following more general system which contains (1.5) as a special case.

3.1 $$\begin{aligned} {\left\{ \begin{array}{ll} u_t-du_{xx}=f(t,x, u), & t>0,\; g(t)<x<h(t),\\ u(t,g(t))=u(t,h(t))=\delta , & t>0,\\ g'(t)=-\frac{d}{\delta }u_{x}(t,g(t)), & t>0,\\ h'(t)=-\frac{d}{\delta }u_x(t,h(t)), & t>0,\\ -g(0)=h(0)=h_0, u(0,x)=u_0(x), & -h_0\le x\le h_0. \end{array}\right. } \end{aligned}$$

For the local existence and uniqueness result, we only require $\delta >0$ and

3.2 $$\begin{aligned} {\left\{ \begin{array}{ll} \ f \in C^{\beta /2, \beta , 1}([0, \infty )\times {\mathbb {R}} \times [0, \infty )) \text{ for } \text{ some } \beta \in (0,1), f(t,x,0) \equiv 0, \text{ and } \text{ for } \text{ any } M>0,\\ f(t,x,u) \text{ is } \text{ Lipschitz } \text{ in } u\in [0, M] \text{ uniformly } \text{ with } \text{ respect } \text{ to } t\ge 0 \text{ and } x\in {\mathbb {R}}. \end{array}\right. } \end{aligned}$$

For global existence and uniqueness, we will further require

3.3 $$\begin{aligned} f(t,x,u)<0 \text{ for } \text{ all } \text{ large } u\text{, } \text{ say } u\ge M_0\text{, } \text{ and } \text{ all } t\ge 0, x\in {\mathbb {R}}. \end{aligned}$$

More precisely, we have the following local and global existence results.

### Theorem 3.1

Assuming that (3.2) is satisfied, then for any $u_0 \in X(h_0)$ and $\alpha \in (0, 1)$, there exists a $T > 0$ such that problem (3.1) has a unique solution

$$\begin{aligned} (u, g, h) \in C^{(1+\alpha )/2, 1+\alpha }(\Omega _T) \times \left[ C^{1+\alpha /2}([0, T])\right] ^2. \end{aligned}$$

Moreover,

$$\begin{aligned} \Vert u\Vert _{C^{(1+\alpha )/2, 1+\alpha }(\Omega _T)} + \Vert g\Vert _{C^{1+\alpha /2}([0, T])} + \Vert h\Vert _{C^{1+\alpha /2}([0, T])} \le C, \end{aligned}$$

and

$$\begin{aligned} h, g \in C^{1+(1+\beta )/2}((0, T]), \quad u \in C^{1+\beta /2, 2+\beta }(\Omega _T), \quad u > 0 \text { in } \Omega _T, \end{aligned}$$

where $\beta$ is from (3.2), $\Omega _T = \{(t, x) \in {\mathbb {R}}^2 : t \in (0, T], x \in [g(t), h(t)]\}$, and the constants $C$ and $T$ depend only on $h_0$, $\alpha$ and $\Vert u_0\Vert _{C^2([-h_0, h_0])}$.

### Theorem 3.2

Assuming that (3.2) and (3.3) are satisfied, then the conclusions in Theorem 3.1 hold for any $T>0$.

Further results on the long-time dynamics of (3.1) are obtained for a special class of *f*(*t*, *x*, *u*) with the following properties:

3.4 $$\begin{aligned} {\left\{ \begin{array}{ll} \text{(i) } \text{(3.2) } \text{ holds, } f(t,x,1)\equiv 0,\ f(t,x,u)\le {{\bar{f}}}(u)< 0 \text{ for } t\ge 0, \ x\in {\mathbb {R}} \text{ and } u>1,\\ \qquad \text{ where } {{\bar{f}}}(u) \text{ is } \text{ a } \text{ continuous } \text{ function } \text{ over } [1,\infty ), \\ \text{(ii) } \text{ there } \text{ exists } {\underline{f}}(u) \text{ satisfying }  \mathbf{(f_A)} \text{ such } \text{ that } f(t,x,u)\ge {\underline{f}}(u) \text{ for } t\ge 0,\ x\in {\mathbb {R}},\ u\ge 0. \end{array}\right. } \end{aligned}$$

Clearly any *f*(*u*) of type $\mathbf{(f_A)}$ satisfies (3.4). Let us note that any monostable *f*(*u*) also satisfies (3.4) with some ${\underline{f}}(u)$ of type $\mathbf{(f_A)}$ with $\theta =\theta ^*_{{\underline{f}}}=0$.

### Theorem 3.3

Suppose that (3.4) holds and $\delta \in (\theta _{{\underline{f}}}^*, 1)$. Then for every initial function $u_0 \in X(h_0)$, (3.1) has a unique solution (*u*(*t*, *x*), *g*(*t*), *h*(*t*)) defined for all $t>0$. Moreover, as $t \rightarrow \infty$,

$$\begin{aligned} (g(t), h(t)) \rightarrow (-\infty , \infty ), \quad u(t, x) \rightarrow 1 \quad \text {locally\ uniformly\ in }\ x \in {\mathbb {R}}. \end{aligned}$$

Furthermore, there exists a constant $M_0>0$ such that

$$\begin{aligned}&{[}g(t), h(t)]\supset [-{\underline{c}}^* t+M_0, \ {\underline{c}}^* t- M_0] \text{ for } \, t>0, \\&\lim _{t \rightarrow \infty } u(t, x) = 1 \quad \text{uniformly\ for } x\in [-({\underline{c}}^*-\epsilon )t, ({\underline{c}}^*-\epsilon )t] \text{ for\ any\ small } \epsilon >0, \end{aligned}$$

where ${\underline{c}}^*>0$ is the unique speed of the semi-wave problem (1.6) with *f* replaced by ${\underline{f}}$.

When $\delta >1$, the vanishing behaviour described in Conjecture 1 is a consequence of the following more general result.

### Theorem 3.4

Suppose that $\delta \in (1,\infty )$, part (i) of (3.4) holds and additionally *f*(*t*, *x*, *u*) is uniformly $C^{\beta /2}$-continuous in *t* for $t\ge 0$, $x\in {\mathbb {R}}$ and $u\in [0,\delta ]$. Then for every initial function $u_0 \in X(h_0)$, vanishing happens to (3.1), namely, there exists $\xi ^*\in {\mathbb {R}}$ depending on *f* and $u_0$ such that

$$\begin{aligned} \lim _{t\rightarrow \infty } g(t)=\lim _{t\rightarrow \infty } h(t)=\xi ^*, \ \lim _{t\rightarrow \infty } u(t,x)=\delta\ \text{ uniformly }\ \text{ for }\ x\in [g(t), h(t)]. \end{aligned}$$

When $\delta =1$, the dynamics of (3.1) exhibits a transition behaviour between successful spreading (as in Theorem 3.3) and vanishing (as in Theorem 3.4), which is described by the following theorem.

### Theorem 3.5

Suppose that (3.4) holds and $\delta =1$. Then for every initial function $u_0 \in X(h_0)$, the unique solution (*u*(*t*, *x*), *g*(*t*), *h*(*t*)) of (3.1) satisfies, as $t \rightarrow \infty$,

$$\begin{aligned}{\left\{ \begin{array}{ll} {[}g(t), h(t)] \rightarrow [g_\infty , h_\infty ]\ \text{ is }\ \text{ a }\ \text{ finite }\ \text{ interval, } \\ \ u(t, x) \rightarrow 1 \quad \text { uniformly\ for }\ x \in [g(t), h(t)]. \end{array}\right. } \end{aligned}$$

Moreover, $h_\infty -g_\infty >0$ always holds except that, in the case $u_0(x)-1$ changes sign in $[-h_0, h_0]$, we need to assume additionally that there exists some small $\varepsilon >0$ such that

3.5 $$\begin{aligned} \varepsilon {\underline{f}}(u)\ge {{\bar{f}}}(u) \text{ for } u\in [1, 1+\varepsilon ]. \end{aligned}$$

The additional condition (3.5) is assumed for technical reasons, which we believe is not necessary. It should be noted that both ${\underline{f}}(u)$ and ${{\bar{f}}}(u)$ are negative for $u>1$, and (3.5) is not needed if $f=f(u)$ is independent of (*t*, *x*) and is non-increasing near $u=1$.

The proofs of the theorems in this section are postponed to Sect. 5.

## Discussions

The main finding of this work is contained in Theorem 1.1, which states that an invasive species can always successfully invade the new territory if it maintains its density level at the invading front at a suitable level by advancing or retreating its range boundary. Moreover, the invasion exhibits an asymptotic spreading speed, with the front location as well as the density distribution determined by an associated semi-wave problem for all large time. This is based on a simple free boundary model (1.5) first considered in Du (2024) for a monostable growth term *f*(*u*). Here we consider more general types of *f*(*u*), including in particular the bistable growth terms, which have been widely used to capture the Allee effect. Indeed, in the classical models with such *f* (Aronson and Weinberger 1975, 1978; Fife and McLeod 1977), as well as in the more recent free boundary models of Du and Lin (2010), Du and Lou (2015) and related works, bistable *f* always leads to vanishing of the species when the initial population is small. Our model (1.5) thus exhibits a new phenomenon, where Allee effect is eliminated. On the other hand, the spreading profile being determined by an associated semi-wave problem is analogous to that already known in earlier models.

The other new feature of (1.5) is that the population range may expand or shrink as time increases, which is strikingly different from earlier free boundary models in Du and Lin (2010), Du and Lou (2015) and related works, where the population range always expands as time increases. If the density level $\delta$ maintained at the range boundary is above the carrying capacity of the environment (i.e., $\delta >1$), then our Theorem 3.4 shows that the species will vanish by the population range shrinking to one point as time goes to infinity. If $\delta$ equals the carrying capacity ($\delta =1$), then Theorem 3.5 reveals that, as time increases to infinity, the population range will converge to a finite interval and the population density will stabilise at the carrying capacity 1 everywhere in the population range. These results suggest that for the invasive species to spread successfully, the density level at the range boundary should be kept at a suitable level, definitely below the carrying capacity of the environment. On the other hand, Theorem 3.3 gives a lower bound $\theta ^*_f\in (0,1)$ for the front density, so that for any $\delta \in (\theta ^*_f, 1)$, successful invasion is guaranteed. For $\delta \in (0, \theta ^*_f)$, the long-time dynamics of (1.5) could be rather varied according to our numerical simulations in Sect. 2, but we have no definite theoretical results for this case so far, except for combustion type of *f* in Conjecture 4 part (i).

As our results in Sect. 3 indicate, the phenomena described in biological terms in the previous paragraph for (1.5) remain valid for the more general model (3.1), where heterogeneous environment is included. This suggests that these phenomena are robust, retained under perturbations of environmental non-homogeneity.

We would like to add some words of caution on the biological interpretations given above based on (1.5) and (3.1), since biological invasion is a very complex process, but in these models many realistic factors have been ignored in order to allow in-depth mathematical analysis. A well known difficulty in biological modelling is the lack of first principles. Unlike many models in the physical science which are based on widely accepted laws, biological models are mostly based on plausible assumptions. As biological processes are usually very complex, affected by multiple factors, such as habitat heterogeneity, predation, competition, and stochastic effects (Lewis et al. 2016), it always carries a high risk to interpret the mathematical results from a model made simple to avail powerful mathematical techniques. On the other hand, new phenomena from such simple models may shed useful insights in the understanding of the complex biological processes being considered.

In the case of (1.5), we have restricted to a single species, and followed a widely used reaction–diffusion equations approach, but modified an existing free boundary condition (the Stefan condition) to the current form (following Du 2024), which can be deduced from the biological assumption that the species advance or retreat the front by maintaining a preferred density there. This new model allows the population range to expand as well as to shrink, which is more natural, and agrees with observations of the spreading of cane toads in Australia (Macgregor et al. 2021), where the total area of cane toads in New South Wales during the period 1971–2020 experienced gradual reduction (in the years 1971-2000), followed by expansion (in 2001-2020); see Fig. 3 in Macgregor et al. (2021).

We hope our mathematical results, with caution, may shed some lights on the understanding of biological invasion in the real world, and motivate further work in this direction. In particular, we would like to know whether any species in the real world indeed uses the strategy at the range boundary suggested by (1.5) to eliminate the Allee effect and evolve into a super invader. Would the Australian cane toads be a candidate? In future work we would like to add more realistic factors to this model, and examine the effect of heterogeneous environment, the presence of other species as competitors or predators, etc.

In a different direction, several recent works explore the effect of trait evolution on the propagation behaviour of invasive species. The observation that toads at the front have longer legs and move faster (Phillips et al. 2006) is an example of non-uniform space-trait distribution in an invasive population. Therefore it is natural to assume that the population density function *u* depends on a certain trait denoted by $\theta$, apart from its dependence on time *t* and spatial location *x*. Such an approach was taken in several recent works; see, for example, Bouin and Henderson (2017), Bouin et al. (2017), Bouin et al. (2018), Alfaro et al. (2021).

More precisely, the model in Bouin and Henderson (2017) has the form

4.1 $$\begin{aligned} u_t=\theta u_{xx}+u_{\theta \theta }+f(u)\ \ \text{ for } t>0,\ x\in {\mathbb {R}},\ \theta \ge {\bar{\theta }}, \end{aligned}$$

where *f*(*u*) is a bistable function, and ${\bar{\theta }}>0$ is a constant. So in this model the variable $\theta$ stands for a trait that represents the spatial dispersal rate of the species. In Bouin et al. (2017), a special monostable *f*(*u*) is used, and both local and nonlocal crowding effects are considered; more precisely, the following model is analysed for local crowding:

4.2 $$\begin{aligned} u_t=\theta u_{xx}+u_{\theta \theta }+u(1-u)\ \ \text{ for } t>0,\ x\in {\mathbb {R}},\ \theta \ge {\bar{\theta }}, \end{aligned}$$

and for nonlocal crowding, (4.2) is modified to

4.3 $$\begin{aligned} u_t=\theta u_{xx}+u_{\theta \theta }+u(1-\int _{{\bar{\theta }}}^\infty ud\theta )\ \ \text{ for } t>0,\ x\in {\mathbb {R}},\ \theta \ge {\bar{\theta }}. \end{aligned}$$

A common feature shared by (4.1), (4.2) and (4.3) is that the species propagates in space with a superlinear rate in time, of the order $t^{3/2}$ as $t\rightarrow \infty$; see Bouin and Henderson (2017), Bouin et al. (2017) for details, and also see Bouin et al. (2018) for a variation of (4.2) where the intrinsic growth rate 1 is replaced by $1-m(\theta )$.

In Alfaro et al. (2021), a bistable growth function was used with the trait $\theta$ representing the Allee threshold level, which varies over a finite interval $(\theta _*, \theta ^*)$ in ${\mathbb {R}}$, and the model has the form

4.4 $$\begin{aligned} u_t=d u_{xx}+\alpha u_{\theta \theta }+u(\int _{\theta _*}^{\theta ^*} ud\theta -\theta )(1-\int _{\theta _*}^{\theta ^*} ud\theta ) \ \ \text{ for } t>0,\ x\in {\mathbb {R}},\ \theta \in (\theta _*,\theta ^*). \end{aligned}$$

Here *d* and $\alpha$ are fixed positive constants. Interesting dynamical behaviour of (4.4) was established in Alfaro et al. (2021), although many questions remain to be answered.

It would be interesting to include a trait factor in our free boundary models considered in this paper.

Several other variations of the free boundary model (1.4) have been considered in recent years for various purposes, and we refer to Bao et al. (2018), Cai et al. (2014), Feng et al. (2022), Kaneko et al. (2020) for a small sample. See also Basiri et al. (2021), Basiri et al. (2025), Lutscher et al. (2020) for rather different free boundary models for the evolution of range boundary. However, these are all for very different purposes from the one in this paper.

## Data Availability

Data sharing is not applicable to this article as no datasets are generated or analysed during the current study.

## 5 Appendix: Proofs of the theorems in Sect. 3 and Conjecture 4 part (i)

This section is organised as follows. In Sect. 5.1, we prove the local well-posedness of (3.1). In Sect. 5.2, we present some comparison principles and obtain several crucial a priori bounds for the local solution of (3.1), which will be used in the proof of the global existence and uniqueness result for (3.1) in Sect. 5.3. Section 5.4 is devoted to the proof of Theorem 3.3 and Sect. 5.5 gives the proof of Theorem 3.4. In Sect. 5.6, we prove Conjecture 4 part (i) and Theorem 3.5.

### 5.1 Proof of the local existence and uniqueness theorem

#### Proof of Theorem 3.1

The strategy of the proof is as follows. For any given continuous function pair (*g*(*t*), *h*(*t*)), $0\le t\le T$, in a suitable set, say $B^T$, and any continuous function *u*(*t*, *x*), $0\le t\le T, x\in [g(t), h(t)]$, in a certain set $Z^T$, we solve the initial boundary value problem

5.1 $$\begin{aligned} {\left\{ \begin{array}{ll} v_t-dv_{xx}=f(t,x, u), & 0<t\le T,\; g(t)<x<h(t),\\ v(t,g(t))=v(t,h(t))=\delta , & 0<t\le T,\\ -g(0)=h(0)=h_0, v(0,x)=u_0(x), & -h_0\le x\le h_0. \end{array}\right. } \end{aligned}$$

The solution $v(t,x)=v^{(u, g,h)}(t,x)$ to (5.1) depends on (*u*, *g*, *h*) and induces a mapping

5.2 $$\begin{aligned} (u(t,x), g(t), h(t))\rightarrow (v(t,x), -h_0-\int _0^t \frac{d}{\delta }v_x(s, g(s))ds, h_0-\int _0^t\frac{d}{\delta }v_x(s, h(s))ds). \end{aligned}$$

If $(u^*, g^*, h^*)$ is a fixed point of the above mapping, then it is easy to see that $(u^*(t,x), g^*(t), h^*(t))$ solves (3.1) for $0\le t\le T$. We will show that the above mapping is indeed a contraction mapping over a suitable set of (*u*, *g*, *h*) when *T* is sufficiently small (and hence has a unit fixed point), which relies on the applications of parabolic $L^p$ estimates and Sobolev embeddings.

This strategy is carried out below in four steps. In Step 1, we show that for small $T>0$, (5.1) can be changed to an equivalent initial boundary value problem with straight lateral boundaries, for which existing parabolic $L^p$ estimates can be readily applied. But to control the desired norm of the solution by the Sobolev embeddings in order to apply the contraction mapping theorem later, we encounter a problem: the embedding results involve a constant which depends on *T* and to obtain a contraction mapping we require this constant to be independent of *T* for all small *T*. To overcome this difficulty we will use an extension trick, which is given in Step 2. Using this trick, we are able to prove the existence of a unique solution to the straight boundary problem obtained in Step 1 and show that for all small $T>0$, the mapping in (5.2), but with *v* replaced by the solution *U* of the equivalent straight boundary problem of Step 1, is a contraction mapping, and hence has a unique fixed point; these constitute Step 3, which yields a solution to (3.1) for $0\le t\le T$ with sufficiently small $T>0$. Finally in Step 4, we explain that the obtained solution has enough regularity to be regarded as a classical solution.

**Step 1**: Straightening the boundaries.

We transform the curved boundaries of (5.1) into straight boundaries. For a given pair $(g(t), h(t))$, which are continuous in *t* with $(g(0), h(0)) = (-h_0, h_0)$, we select a function $\zeta \in C^3({\mathbb {R}})$ such that

$$\begin{aligned} \zeta (y) = {\left\{ \begin{array}{ll} 1, & \text {if } |y - h_0|< \frac{h_0}{4}, \\ 0, & \text {if } |y - h_0| > \frac{h_0}{2}, \end{array}\right. } \quad \text {and } |\zeta '(y)| < \frac{5}{h_0} \text { for all } y. \end{aligned}$$

Define $\xi (y) = -\zeta (-y)$, and introduce the transformation $(t, y) \mapsto (t, x)$ given by

$$\begin{aligned} x = \Psi (t, y):= y + \zeta (y)(h(t) - h_0) - \xi (y)(g(t) + h_0) \quad \text {for } y \in {\mathbb {R}}. \end{aligned}$$

This transformation is a diffeomorphism for fixed $t \in [0, T]$ from $[-h_0, h_0]$ to [*g*(*t*), *h*(*t*)], provided that $T>0$ is small such that $|h(t) - h_0| < \frac{h_0}{12}$ and $|g(t) + h_0| < \frac{h_0}{12}$ for $t\in [0, T]$. Under the inverse of this transformation, it is clear that the curved boundaries $x = g(t)$ and $x = h(t)$ are mapped to $y = -h_0$ and $y = h_0$, respectively.

Direct calculations yield the following expressions:

5.3 $$\begin{aligned} {\left\{ \begin{array}{ll} \displaystyle \frac{\partial y}{\partial x} = \frac{1}{1 + \zeta '(y)(h(t) - h_0) - \xi '(y)(g(t) + h_0)} =: \sqrt{A(y, h(t), g(t))}, \\ \displaystyle \frac{\partial ^2 y}{\partial x^2} = \frac{-\zeta ''(y)(h(t) - h_0) - \xi ''(y)(g(t) + h_0)}{\left[ 1 + \zeta '(y)(h(t) - h_0) - \xi '(y)(g(t) + h_0)\right] ^3} =: B(y, h(t), g(t)), \\ \displaystyle \frac{\partial y}{\partial t} = \frac{-h'(t)\zeta (y) + g'(t)\xi (y)}{1 + \zeta '(y)(h(t) - h_0) - \xi '(y)(g(t) + h_0)} \\ \ \ \ \ \ =: -h'(t)C(y, h(t), g(t)) + g'(t)D(y, h(t), g(t)). \end{array}\right. } \end{aligned}$$

Define $U(t, y):= v(t, x)$ for $(t, x) \in \Omega _T$. Then (5.1) is reduced to

5.4 $$\begin{aligned} {\left\{ \begin{array}{ll} U_t - dA U_{yy} - (h'C - g'D + d B) U_y = f(t, \Psi (t, y),u(t, \Psi (t, y)), & 0< t \le T, \, -h_0< y< h_0,\\ U(t, h_0) = U(t, -h_0) = \delta , & 0 < t \le T,\\ h(0) = h_0, \quad g(0) = -h_0, \quad U(0, y) = u_0(y), \quad & -h_0 \le y \le h_0. \end{array}\right. } \end{aligned}$$

If *U* solves (5.4), then through the transformation $\Psi$ we can obtain

$$\begin{aligned} v(t,x)=U(t, \Psi ^{-1}(t, x)), \end{aligned}$$

which solves (5.1). Here $\Psi ^{-1}(t,\cdot )$ is the inverse of $\Psi (t,\cdot )$, i.e.,

$$\begin{aligned} y=\Psi ^{-1}(t, \Psi (t,y)) \text{ for } t\in [0, T],\ y\in [-h_0, h_0]. \end{aligned}$$

**Step 2**: An extension trick, and application of $L^p$ theory and Sobolev embeddings.

Define $$T_1$$ as

$$\begin{aligned} T_1:= \min \left\{ \frac{h_0}{12(1+h^0)}, \frac{h_0}{12(1-g^0)}\right\} , \end{aligned}$$

where $h^0 = -\frac{d}{\delta }U_y(0, h_0)$ and $g^0 = -\frac{d}{\delta }U_y(0, -h_0)$. For $T \in (0, T_1]$, let

$$\begin{aligned} D_T:= \{(t, y): 0 \le t \le T, -h_0 \le y \le h_0\}. \end{aligned}$$

We now introduce the following function spaces:

$$\begin{aligned}&X_{1, T} := \{U \in C(D_T) : U(0, y) = u_0(y), \Vert U - u_0\Vert _{C(D_T)} \le 1\},\\&X_{2, T} := \{h \in C^1([0, T]) : h(0) = h_0, h'(0) = h^0, \Vert h' - h^0\Vert _{C([0, T])} \le 1\},\\&X_{3, T} := \{g \in C^1([0, T]) : g(0) = -h_0, g'(0) = g^0, \Vert g' - g^0\Vert _{C([0, T])} \le 1\}. \end{aligned}$$

Clearly, the product space $X_T:= \prod _{i=1}^3 X_{i, T}$ is a complete metric space when equipped with the metric:

$$\begin{aligned} d((U_1, h_1, g_1), (U_2, h_2, g_2)):= \Vert U_1 - U_2\Vert _{C(D_T)} + \Vert h_1' - h_2'\Vert _{C([0, T])} + \Vert g_1' - g_2'\Vert _{C([0, T])}. \end{aligned}$$

For $0< T < T_1$, we define a subspace of $X_{T_1}$, denoted by $X_{T_1}^T:= \prod _{i=1}^3 X_{i, T_1}^T$, as follows

$$\begin{aligned}&X_{1,T_1}^{T} := \{U \in X_{1, T_1} : U(t,y) = U(T, y) \text { for } T \le t \le T_1\},\\&X_{2,T_1}^{T} := \{h \in X_{2, T_1} : h(t) = h(T)+h'(T)(t-T) \text { for } T \le t \le T_1\},\\&X_{3,T_1}^{T} := \{g \in X_{3, T_1} : g(t) = g(T)+g'(T)(t-T) \text { for } T \le t \le T_1\}. \end{aligned}$$

Thus, for each $(U, h, g) \in X_T$, we can extend it to a member of $X_T^{T_1}$. Henceforth, we will identify $X_T$ with $X_T^{T_1}$.

For $(U, h, g) \in X_T = X^T_{T_1} \subseteq X_{T_1}$, using the expressions for *A*, *B*, and *C* from (5.3), we obtain the following estimate

5.5 $$\begin{aligned} \begin{aligned} \Vert h'C - g'D + dB\Vert _{C(D_{T_1})} \le \sup _{(y, t) \in D_{T_1}} \frac{|h'(t)\zeta (y) - g'(t)\xi (y)|}{1 + \zeta '(y)(h(t) - h_0) - \xi '(y)(g(t) + h_0)} \\ + d \sup _{(y, t) \in D_{T_1}} \frac{|\zeta ''(y)(h(t) - h_0) + \xi ''(y)(g(t) + h_0)|}{\left[ 1 + \zeta '(y)(h(t) - h_0) - \xi '(y)(g(t) + h_0)\right] ^3} \le L_1, \end{aligned} \end{aligned}$$

where $L_1$ depends on $h_0$, $h^0$, and $g^0$. Furthermore, for $(t, y) \in D_{T_1}$, it is straightforward to verify

5.6 $$\begin{aligned} \frac{d}{4} \le dA(y, h(t), g(t)) \le 36d. \end{aligned}$$

For two points $Y_1 = (s_1, y_1)$ and $Y_2 = (s_2, y_2)$ in $D_{T_1}$, with parabolic distance

$$\begin{aligned} \delta (Y_1, Y_2) = \sqrt{(y_1 - y_2)^2 + |s_1 - s_2|}, \end{aligned}$$

we define

5.7 $$\begin{aligned} \begin{aligned} \omega (R):=&\ d \max _{Y_1, Y_2 \in D_{T_1}} \sup _{\delta (Y_1, Y_2) \le R} \left| A(y_1, h(s_1), g(s_1)) - A(y_2, h(s_2), g(s_2)) \right| \\ \le&\ 1296 \, d \bigg | {\left( 1 + \zeta '(y_2)(h(s_2) - h_0) - \xi '(y_2)(g(s_2) + h_0) \right) ^2}\\&\hspace{1.2cm} - {\left( 1 + \zeta '(y_1)(h(s_1) - h_0) - \xi '(y_1)(g(s_1) + h_0) \right) ^2} \bigg | \\ \le&C\left( |\zeta '(y_2)-\zeta '(y_1)|+|\xi '(y_2)-\xi '(y_1)|+|g(s_2)-g(s_1)|+|h(s_2)-h(s_1)|\right) \\ \le&\ l_1 R \rightarrow 0 \quad \text {as } R \rightarrow 0, \end{aligned} \end{aligned}$$

where *C* and $l_1$ are some suitably large constant depending on $h_0$, $h^0$, and $g^0$.

For (*u*, *g*, *h*) in (5.1), we write $U(t,y):=u(t,\Psi ^{-1}(t,y))$ and consider the initial boundary value problem

5.8 $$\begin{aligned} {\left\{ \begin{array}{ll} {\bar{U}}_t - dA {\bar{U}}_{yy} - (h'C - g'D + d B){\bar{U}}_y = f(t, \Psi ^{-1}(t,y),U), & -h_0< y< h_0, \, 0< t \le T_1, \\ {\bar{U}}(t, h_0) = {\bar{U}}(t, -h_0) = \delta , & 0 < t \le T_1, \\ {\bar{U}}(0, y) = u_0(y), & -h_0 \le y \le h_0. \end{array}\right. } \end{aligned}$$

Using $L^p$ theory and the Sobolev embedding theorem, we conclude that (5.8) has a unique solution ${\bar{U}}$ satisfying

5.9 $$\begin{aligned} \Vert {\bar{U}}\Vert _{C^{(1+\alpha )/2, 1+\alpha }(D_{T_1})} \le C_{T_1} \Vert {\bar{U}}\Vert _{W^{2,1}_p(D_{T_1})} \le K_1, \end{aligned}$$

where $$p > $$$$ 3/(2 - \alpha )$$, and $$K_1$$ depends on *p*, $$\Vert f(\cdot , \cdot , U(\cdot ,\cdot ))$$$$\Vert _{L^p(D_{T_1})}$$, $$\Vert u_0\Vert _{C^2([-h_0, h_0])}$$, $$L_1$$, $$D_{T_1}$$, $$l_1$$, and $$C_{T_1}$$ depends on $$D_{T_1}$$ and $$\alpha \in (0,1)$$.

Define ${\bar{h}}(t)$ and ${\bar{g}}(t)$ by ${\bar{h}}'(t) = -\frac{d}{\delta } {\bar{U}}_y(t, h_0)$, ${\bar{g}}'(t) = -\frac{d}{\delta } {\bar{U}}_y(t, -h_0)$. It is clear that ${\bar{h}}', {\bar{g}}' \in C^{\alpha /2}([0, T_1])$ and satisfy

5.10 $$\begin{aligned} \Vert {\bar{h}}'\Vert _{C^{\alpha /2}([0, T_1])}, \Vert {\bar{g}}'\Vert _{C^{\alpha /2}([0, T_1])} \le K_2, \end{aligned}$$

where $K_2$ depends on $K_1$.

Now define the mapping $F: X_T = X_T^{T_1} \rightarrow C(D_{T_1}) \times \left[ C([0, T_1])\right] ^2$ by $F(U, h, g) = ({\bar{U}}, {\bar{h}}, {\bar{g}})$, and then consider its restriction on $X_T$, namely

$$\begin{aligned} \tilde{F}(U, h, g):= F(U, h, g)\big |_{X_T}. \end{aligned}$$

We observe that (*U*(*t*, *y*), *h*(*t*), *g*(*t*)) is a fixed point of the mapping $\tilde{F}$ if and only if $(U(t, \Psi ^{-1}(t, x)), h(t), g(t))$ solves (3.1) for $t \in [0, T]$.

**Step 3:** We show that $\tilde{F}$ is a contraction mapping when $T > 0$ is sufficiently small.

For a fixed $0< T < \min \{T_1, K_1^{-\frac{2}{1+\alpha }}, K_2^{-\frac{2}{\alpha }}\}$, we have

$$\begin{aligned}&\Vert {{\bar{U}}} - u_0\Vert _{C(D_T)} \le T^{\frac{1+\alpha }{2}} \Vert {{\bar{U}}}\Vert _{C^{0, (1+\alpha )/2}(D_T)} \le T^{\frac{1+\alpha }{2}} \Vert U\Vert _{C^{0, (1+\alpha )/2}(D_{T_1})} \le K_1 T^{\frac{1+\alpha }{2}} \le 1,\\&\Vert {{\bar{h}}}'(t) - h^0\Vert _{C([0, T])} \le T^{\frac{\alpha }{2}} \Vert {\bar{h}}'\Vert _{C^{\alpha /2}([0, T_1])} \le T^{\frac{\alpha }{2}} \Vert {\bar{h}}'\Vert _{C^{\alpha /2}([0, T_1])} \le K_2 T^{\frac{\alpha }{2}} \le 1,\\&\Vert {{\bar{g}}}'(t) - g^0\Vert _{C([0, T])} \le T^{\frac{\alpha }{2}} \Vert {\bar{g}}'\Vert _{C^{\alpha /2}([0, T_1])} \le T^{\frac{\alpha }{2}} \Vert {\bar{g}}'\Vert _{C^{\alpha /2}([0, T_1])} \le K_2 T^{\frac{\alpha }{2}} \le 1. \end{aligned}$$

These inequalities confirm that $\tilde{F}$ maps $X_T$ into itself.

We now proceed to show that $\tilde{F}$ is a contraction on $X_T$ for small enough $T > 0$. Let $(U_i, h_i, g_i) \in X_T = X_T^{T_1}$ for $i = 1, 2$, and define $W:= {\bar{U}}_1 - {\bar{U}}_2$. Then *W* satisfies the following system

5.11 $$\begin{aligned} {\left\{ \begin{array}{ll} W_t - dA_1 W_{yy} - (dB_1 + h_1'C_1 - g_1'D_1 ) W_y = \Phi , & -h_0< y< h_0, \, 0< t \le T_1, \\ W(t, h_0) = W(t, -h_0) = 0, & 0 < t \le T_1, \\ W(0, y) = 0, & -h_0 \le y \le h_0, \end{array}\right. } \end{aligned}$$

where

$$\begin{aligned} \Phi =&(dB_1 - dB_2 + h_1'C_1 - h_2'C_2 - g_1'D_1+ g_2'D_2) {\bar{U}}_{2, y} \\&+ (dA_1 - dA_2) {\bar{U}}_{2, yy} + f(x,t,U_1) - f(x,t,U_2). \end{aligned}$$

Here, $A_i:= A(y, h_i, g_i)$, $B_i:= B(y, h_i, g_i)$, $C_i:= C(y, h_i, g_i)$, and $D_i:= D(y, h_i, g_i)$ for $i = 1, 2$. A direct calculation yields

$$\begin{aligned}&\Vert A_1 - A_2\Vert _{C(D_{T_1})} = \sup _{(t,y) \in D_{T_1}} \left| A(y, h_1(t), g_1(t)) - A(y, h_2(t), g_2(t)) \right| \\ =&\sup _{(t,y) \in D_{T_1}} \bigg | \frac{1}{\left( 1 + \zeta '(y)(h_1(t) - h_0) - \xi '(y)(g_1(t) + h_0)\right) ^2}\\&\quad \quad \quad \ \ - \frac{1}{\left( 1 + \zeta '(y)(h_2(t) - h_0) - \xi '(y)(g_2(t) + h_0)\right) ^2} \bigg | \\&\le S_1 \left( \Vert h_1 - h_2\Vert _{C([0, T_1])} + \Vert g_1 - g_2\Vert _{C([0, T_1])} \right) , \end{aligned}$$

where $S_1$ is a constant depending on $h_0$, $h^0$, and $g^0$, but independent of $T_1$. Similarly, we deduce

$$\begin{aligned}&\Vert B_1 - B_2\Vert _{C(D_{T_1})}, \, \Vert C_1 - C_2\Vert _{C(D_{T_1})}, \, \Vert D_1 - D_2\Vert _{C(D_{T_1})} \\ \le&\ S_2 \left( \Vert h_1 - h_2\Vert _{C([0, T_1])} + \Vert g_1 - g_2\Vert _{C([0, T_1])} \right) , \end{aligned}$$

where $S_2$ depends similarly on parameters. Moreover, by (5.3),

$$\begin{aligned} \Vert C_2\Vert _{C(D_{T_1})} + \Vert D_2\Vert _{C(D_{T_1})} \le 12 =: S_3. \end{aligned}$$

By combining these estimates with (5.9), we deduce that for sufficiently small $T > 0$,

5.12 $$\begin{aligned} \begin{aligned} \Vert \Phi \Vert _{L^p(D_{T_1})} \le&\ \Vert dB_1 - dB_2 + h_1'C_1 - h_2'C_2 - g_1'D_1 + g_2'D_2\Vert _{L^\infty (D_{T_1})} \Vert {\bar{U}}_{2,y}\Vert _{L^p(D_{T_1})} \\&+ d\Vert A_1 - A_2\Vert _{L^\infty (D_{T_1})} \Vert {\bar{U}}_{2,yy}\Vert _{L^p(D_{T_1})} + \Vert f(\cdot ,\cdot ,U_1) - f(\cdot ,\cdot ,U_2)\Vert _{L^p(D_{T_1})}\\ \le&\ S_1^* \left( \Vert U_1 - U_2\Vert _{C(D_{T_1})} + \Vert h_1 - h_2\Vert _{C^1([0,T_1])} + \Vert g_1 - g_2\Vert _{C^1([0,T_1])} \right) , \end{aligned} \end{aligned}$$

where $S_1^*$ depends on $D_{T_1}$, $K_1$, *f*, and $S_i$ for $i = 1, 2$.

Using (5.5), (5.6), (5.7), and (5.12), we can apply the $L^p$ estimates to (5.11) and the Sobolev embedding theorem to derive

$$\begin{aligned}&\Vert W\Vert _{C^{1+\alpha /2, 1+\alpha }(D_{T_1})} \le C_{T_1} \Vert W\Vert _{W_p^{1,2}(D_{T_1})}\\ \le&K_3 \left( \Vert U_1 - U_2\Vert _{C(D_{T_1})} + \Vert h_1 - h_2\Vert _{C^1([0, T_1])} + \Vert g_1 - g_2\Vert _{C^1([0, T_1])} \right) , \end{aligned}$$

where $K_3$ is determined by *p*, $D_{T_1}$, $S_1^*$, $L_1$, $l_1$, and $C_{T_1}$. Furthermore, from the definitions of ${\bar{h}}_i$ and ${\bar{g}}_i$, we obtain

$$\begin{aligned}&\Vert {\bar{h}}_1' - {\bar{h}}_2'\Vert _{C^{\alpha /2}([0, T_1])} \le \frac{d}{\delta } \Vert {\bar{U}}_{1, y}(\cdot , h_0) - {\bar{U}}_{2, y}(\cdot , h_0)\Vert _{C^{0, \alpha /2}(D_{T_1})},\\&\Vert {\bar{g}}_1' - {\bar{g}}_2'\Vert _{C^{\alpha /2}([0, T_1])} \le \frac{d}{\delta } \Vert {\bar{U}}_{1, y}(\cdot , -h_0) - {\bar{U}}_{2, y}(\cdot , -h_0)\Vert _{C^{0, \alpha /2}(D_{T_1})}. \end{aligned}$$

Then combining the above estimates, we conclude

$$\begin{aligned}&\Vert {{\bar{U}}}_1 - {{\bar{U}}}_2\Vert _{C^{(1+\alpha )/2, 1+\alpha }(D_{T_1})} + \Vert {\bar{h}}_1' - {\bar{h}}_2'\Vert _{C^{\alpha /2}([0, T_1])} + \Vert {\bar{g}}_1' - {\bar{g}}_2'\Vert _{C^{\alpha /2}([0, T_1])}\\ \le&\ K_4 \left( \Vert U_1 - U_2\Vert _{C(D_{T_1})} + \Vert h_1 - h_2\Vert _{C^1([0, T_1])} + \Vert g_1 - g_2\Vert _{C^1([0, T_1])} \right) , \end{aligned}$$

where $K_4$ depends on *d*, $\delta$, and $K_3$. Finally, setting $T = \min \{ \frac{1}{2}, T_1, K_1^{-\frac{2}{1+\alpha }}, K_2^{-\frac{2}{\alpha }}, (2K_4)^{-\frac{2}{\alpha }} \}$ ensures

$$\begin{aligned}&\Vert {\bar{U}}_1 - {\bar{U}}_2\Vert _{C(D_T)} + \Vert {\bar{h}}_1' - {\bar{h}}_2'\Vert _{C([0, T])} + \Vert {\bar{g}}_1' - {\bar{g}}_2'\Vert _{C([0, T])}\\ \le&\ T^{\frac{1+\alpha }{2}} \Vert {\bar{U}}_1 - {\bar{U}}_2\Vert _{C^{\frac{1+\alpha }{2}, 1+\alpha }(D_{T_1})} + T^{\frac{\alpha }{2}} \Vert {\bar{h}}_1' - {\bar{h}}_2'\Vert _{C^{\frac{\alpha }{2}}([0, T_1])} + T^{\frac{\alpha }{2}} \Vert {\bar{g}}_1' - {\bar{g}}_2'\Vert _{C^{\frac{\alpha }{2}}([0, T_1])}\\ \le&\ \frac{1}{2} \left( \Vert U_1 - U_2\Vert _{C(D_{T_1})} + \Vert h_1' - h_2'\Vert _{C([0, T_1])} + \Vert g_1' - g_2'\Vert _{C([0, T_1])} \right) \\ =&\ \frac{1}{2} \left( \Vert U_1 - U_2\Vert _{C(D_T)} + \Vert h_1' - h_2'\Vert _{C([0, T])} + \Vert g_1' - g_2'\Vert _{C([0, T])} \right) , \end{aligned}$$

proving that $\tilde{F}$ is a contraction mapping on $X_T$. Hence, $\tilde{F}$ has a unique fixed point (*U*, *h*, *g*) in $X_T$. Additionally, by the maximum principle, $U > 0$ in $[0, T] \times [-h_0, h_0]$.

**Step 4:** Finally, we employ Schauder theory to derive additional regularity for the solution of (5.4) in $[-h_0, h_0] \times (0, T]$.

From Step 3, we know that $U \in C^{(1+\alpha )/2, 1+\alpha }(D_T)$ and $h, g \in C^{1+\alpha /2}([0, T])$. Consequently, the coefficients in (5.4) satisfy the following regularity properties

5.13 $$\begin{aligned} dA \in C^{\alpha /2, \alpha }(D_T) \quad \text {and} \quad h'C - g'D + dB \in C^{\alpha /2, \alpha }(D_T). \end{aligned}$$

However, since $u_0 \in C^2([-h_0, h_0])$, the Schauder theory cannot be applied to (5.4) direcly. To address this, we employ a standard cut-off technique. Let $\varepsilon > 0$ be any small constant, and choose $\xi ^* \in C^\infty ([0, T])$ such that

$$\begin{aligned} \xi ^*(t) = {\left\{ \begin{array}{ll} 1, & t \in [2\varepsilon , T], \\ 0, & t \in [0, \varepsilon ]. \end{array}\right. } \end{aligned}$$

Define $\tilde{U}:= U \xi ^*$. Using (5.4) we obtain

5.14 $$\begin{aligned} {\left\{ \begin{array}{ll} \tilde{U}_t = dA \tilde{U}_{yy} + (h'C - g'D + dB)\tilde{U}_y + F(t, y), & -h_0< y< h_0, \, 0< t< T, \\ \tilde{U}(t, h_0) = \tilde{U}(t, -h_0) = \delta \xi ^*(t), & 0< t < T, \\ \tilde{U}(0, y) = 0, & -h_0 \le y \le h_0, \end{array}\right. } \end{aligned}$$

where $$F(t, y) = U \xi _t^* + f(t,y,U) \xi ^*$$.

We may assume $$\alpha $$$$ \ge \beta $$ (where *β* is from (3.2)), and then $$F \in C^{\beta /2, \beta }$$$$([-h_0, h_0] \times [0, T])$$. In view of (5.13) and (5.6), we can apply the Schauder estimates to (5.14) to obtain

$$\begin{aligned} \Vert {U}\Vert _{C^{1+\beta /2, 2+\beta }([-h_0, h_0] \times [2\varepsilon , T])} \le \Vert \tilde{U}\Vert _{C^{1+\beta /2, 2+\beta }([-h_0, h_0] \times [0, T])}\\ \le K \big (\Vert F\Vert _{C^{\beta /2, \beta }([-h_0, h_0] \times [0, T])}+\Vert \delta \xi ^*\Vert _{C^{1+\beta /2}([0, T])}\big ), \end{aligned}$$

where *K* depends on $\varepsilon$, $h_0$, $\beta$, and $L_1$ in (5.5). Since $\varepsilon > 0$ can be arbitrarily small, it follows that $h, g \in C^{1+(1+\beta )/2}((0, T])$ and $u \in C^{1+\beta /2, 2+\beta }(\Omega _T)$. Thus (*u*, *g*, *h*) is a classical solution of (3.1) on $\Omega _T$. $\square$

### 5.2 Comparison principles and a priori bounds

In this subsection, we collect some results which are needed to prove the global existence and uniqueness of solutions to (3.1). We first give some comparison principles in a form that is more convenient to use than (Du 2024, Lemma 2.2, Lemma 2.3).

#### Lemma 5.1

(Comparison Principle 1) Assume (3.2) holds, $$T \in $$$$(0, \infty )$$, $$g_*, {\underline{h}}, $$$${\bar{h}} \in C^1([0, T])$$ with $$g_*(t)<\min $$$$ \{{\underline{h}}(t),{{\bar{h}}}(t)\}$$, $${\underline{u}} \in C^{0,1}$$$$({{\overline{D}}}_T) \cap C^{1, 2}( D_T)$$ with $$D_T = \{(t, x) \in {\mathbb {R}}^2: $$$$ 0< t \le T,\ g_*(t)< x < {\underline{h}}(t)\}$$, $${{\bar{u}}} \in C^{0,1}$$$$({{\overline{E}}}_T) \cap C^{1, 2}(E_T)$$ with $$E_T = \{(t, x) \in {\mathbb {R}}^2: $$$$0< t \le T,\ g_*(t)< x < {\bar{h}}(t)\}$$, and

$$\begin{aligned} {\left\{ \begin{array}{ll} {\underline{u}}_t - d{\underline{u}}_{xx} - f(t,x,{\underline{u}})\le {{\bar{u}}}_t - d{{\bar{u}}}_{xx} - f(t,x,{{\bar{u}}}), & 0< t \le T, \, g_*(t)< x< \min \{{\underline{h}}(t),{\bar{h}}(t)\}, \\ {\underline{u}} (t, g_*(t) \le {{\bar{u}}} (t, g_*(t)),\  & 0 \le t \le T, \\ {{\bar{u}}} = \delta , \, {\bar{h}}'(t) \ge -\frac{d}{\delta }{{\bar{u}}}_x, & 0< t \le T, \, x = {\bar{h}}(t), \\ {\underline{u}} = \delta , \, {\underline{h}}'(t) \le -\frac{d}{\delta }{\underline{u}}_x, & 0< t \le T, \, x = {\underline{h}}(t), \\ t_0\in (0, T] \text{ and } {\underline{h}}(t_0)< {\bar{h}}(t_0) \implies {{\bar{u}}} (t_0, {\underline{h}}(t_0))\ge \delta , & \\ {\underline{h}}(0) < {{\bar{h}}}(0), \, {\underline{u}}(0,x) \le {{\bar{u}}}(0, x), & x \in [g_*(0), {\underline{h}}(0)], \end{array}\right. } \end{aligned}$$

then

$$\begin{aligned} {\underline{h}}(t)< {\bar{h}}(t) \ \textrm{for }\ t \in (0, T], \quad {\underline{u}}(t, x)< {\bar{u}}(t, x)\ \textrm{for }\ t \in (0, T] \ \textrm{and }\ g_*(t)< x < {\underline{h}}(t). \end{aligned}$$

#### Proof

We claim that ${\underline{h}}(t)<{{\overline{h}}}(t)$ for all $t\in (0, T]$. Clearly this is true for small $t>0$ since ${\underline{h}}(0)<{{\bar{h}}}(0)$. If our claim does not hold, then we can find a first $t^*\le T$ such that ${\underline{h}}(t)<{{\overline{h}}}(t)$ for $t\in (0, t^*)$ and ${\underline{h}}(t^*)={{\overline{h}}}(t^*)$. It follows that

5.15 $$\begin{aligned} {\underline{h}}'(t^*)\ge {{\overline{h}}}'(t^*). \end{aligned}$$

We now compare ${\underline{u}}$ and ${{\overline{u}}}$ over the region

$$\begin{aligned} \Omega _{t^*}:=\{(t,x)\in {\mathbb {R}}^2: 0< t\le t^*, g_* (t)< x< {\underline{h}}(t)\}. \end{aligned}$$

The strong maximum principle yields ${\underline{u}}(t,x)<{{\overline{u}}}(t,x)$ in $\Omega _{t^*}$. Hence $w(t,x):={{\overline{u}}}(t,x)-{\underline{u}}(t,x)>0$ in $\Omega _{t^*}$ with $w(t^*, {\underline{h}}(t^*))=0$. It then follows from the Hopf boundary lemma that $w_x(t^*, {\underline{h}}(t^*))< 0$, from which we deduce

$$\begin{aligned} {{\overline{h}}}'(t^*)-{\underline{h}}'(t^*)\ge -\frac{d}{\delta }\Big [ {{\overline{u}}}_x(t^*, {{\overline{h}}}(t^*))-{\underline{u}}_x(t^*, {\underline{h}}(t^*))\Big ]=-\frac{d}{\delta }w_x(t^*, {\underline{h}}(t^*))>0, \end{aligned}$$

which is a contradiction to (5.15). This proves our claim that ${\underline{h}}(t)<\overline{h}(t)$ for all $t\in (0, T]$. We may now apply the usual comparison principle over $\Omega _{T}$ to conclude that ${\underline{u}}< \overline{u}$ in $\Omega _T$. $\square$

#### Lemma 5.2

(Comparison Principle 2) Assume (3.2) holds, $$T \in $$$$(0, \infty )$$, $${\underline{g}}, {\underline{h}}, $$$${{\bar{g}}}, {\bar{h}} \in C^1([0, T])$$ with $$G(t):= \max \{{\underline{g}}(t), {{\bar{g}}}(t)\} $$$$<H(t):= \min \{{\underline{h}}(t),{\bar{h}}(t)\}$$ for $$t\in [0, T]$$, $${\underline{u}} \in C^{0,1}$$$$({{\bar{D}}}_T) \cap C^{1, 2}(D_T)$$ with $$D_T = \{(t, x) \in {\mathbb {R}}^2: $$$$ 0< t \le T,\ {\underline{g}}(t)< x < {\underline{h}}(t)\}$$, $${{\bar{u}}} \in C^{0,1}$$$$({{\bar{E}}}_T) \cap C^{1, 2}(E_T)$$ with $$E_T = \{(t, x) \in {\mathbb {R}}^2: $$$$ 0< t \le T,\ {{\bar{g}}}(t)< x < {\bar{h}}(t)\}$$, and

$$\begin{aligned} {\left\{ \begin{array}{ll} {\underline{u}}_t - d{\underline{u}}_{xx} - f(t,x,{\underline{u}})\le {{\bar{u}}}_t - d{{\bar{u}}}_{xx} - f(t,x,{{\bar{u}}}), & 0< t \le T, \, G(t)< x< H(t), \\ {\underline{u}} = \delta , \, {\underline{g}}'(t) \ge -\frac{d}{\delta }{\underline{u}}_x, & 0< t \le T, \, x = {\underline{g}}(t), \\ {\underline{u}} = \delta , \, {\underline{h}}'(t) \le -\frac{d}{\delta }{\underline{u}}_x, & 0< t \le T, \, x = {\underline{h}}(t), \\ {{\bar{u}}} = \delta , \, {{\bar{g}}}'(t) \le -\frac{d}{\delta }{{\bar{u}}}_x, & 0< t \le T, \, x = {{\bar{g}}}(t), \\ {{\bar{u}}} = \delta , \, {\bar{h}}'(t) \ge -\frac{d}{\delta }{{\bar{u}}}_x, & 0< t \le T, \, x = {\bar{h}}(t), \\ t_0\in (0, T] \text{ and } {\underline{h}}(t_0) < {\bar{h}}(t_0) \implies {{\bar{u}}} (t_0, {\underline{h}}(t_0))\ge \delta , & \\ t_0\in (0, T] \text{ and } {\underline{g}}(t_0) > {{\bar{g}}}(t_0) \implies {{\bar{u}}} (t_0, {\underline{g}}(t_0))\ge \delta , & \\ {[}{\underline{g}}(0), {\underline{h}}(0)]\subset ({{\bar{g}}}(0), {{\bar{h}}}(0)), \, {\underline{u}}(0,x) \le {{\bar{u}}}(0, x), & x \in {[}{\underline{g}}(0), {\underline{h}}(0)], \end{array}\right. } \end{aligned}$$

then

$$\begin{aligned} {[}{\underline{g}}(t), {\underline{h}}(t)]\subset ({{\bar{g}}}(t), {\bar{h}}(t)) \ \textrm{for }\ t \in (0, T], \quad {\underline{u}}(t, x)< {\bar{u}}(t, x)\ \textrm{for }\ t \in (0, T] \ \mathrm{and \ } {\underline{g}}(t)< x < {\underline{h}}(t). \end{aligned}$$

#### Proof

This is a simple variation of the proof of Lemma 5.1, and is left to the interested reader. $\square$

#### Remark 5.3

The functions $({\bar{u}}, {\bar{h}})$ and $({\underline{u}}, {\underline{h}})$ in Lemmas 5.1 are called a pair of upper and lower solutions to (3.1); similarly, the function triples $({\bar{u}}, {{\bar{g}}}, {\bar{h}})$ and $({\underline{u}}, {\underline{g}}, {\underline{h}})$ in Lemma 5.2 are called a pair of upper and lower solutions to problem (3.1). There is a symmetric version of Lemma 5.1, where the conditions on the left and right boundaries are swapped.

The following result indicates that the population range [*g*(*t*), *h*(*t*)] does not shrink to a single point in finite time.

#### Lemma 5.4

Assume (3.2) and (3.3) hold, $T \in (0, \infty )$, and (*u*, *g*, *h*) solves (3.1) for $0< t < T$. Then there exist positive constants $C_1(T)$ and $C_3(T)$ such that

$$\begin{aligned} C_1(T)\le u(t, x)\le C_2, \ h(t)-g(t)\ge C_3(T) \quad \text{ for }\ t \in (0, T),\ x \in [g(t), h(t)], \end{aligned}$$

where $C_2:=\max _{x\in [-h_0,h_0]}{u_0}(x)+M_0$ with $M_0$ given by (3.3).

#### Proof

Consider the initial boundary value problem

5.16 $$\begin{aligned} {\left\{ \begin{array}{ll} v_t - dv_{xx} = f(x,t,v), & t \in (0, T), \, x \in (g(t), h(t)), \\ v(t, g(t)) = v(t, h(t)) = \delta , & t \in (0, T), \\ v(0, x) = v_0, & x \in [-h_0, h_0], \end{array}\right. } \end{aligned}$$

with $v_0=0$ or $v_0=\max _{x\in [-h_0,h_0]}{u_0}(x)+M_0$. Denote by ${\underline{v}}$ the solution of (5.16) with initial condition $v_0=0$, and ${{\bar{v}}}$ the solution of (5.16) with initial condition $v_0=\max _{x\in [-h_0,h_0]}{u_0}(x)+M_0$. By standard comparison principle, we have

$$\begin{aligned} C_2\ge {{\bar{v}}}(t,x)\ge u(t, x) \ge {\underline{v}}(t, x) > 0 \quad \text{ for } t \in (0, T),\ x \in [g(t), h(t)], \end{aligned}$$

with $C_2=\max _{x\in [-h_0,h_0]}{u_0}(x)+M_0$. Then by (3.2), there exists $L > 0$ depending on $C_2$ such that

5.17 $$\begin{aligned} f(x,t,u) \ge -Lu \quad \text{ for } t \in [0, T),\ x \in [g(t), h(t)]. \end{aligned}$$

Let $$v_1(t)$$ be the solution of the ODE problem $$v_1'=-Lv_1$$ with $$v_1(0)=$$$$\min _{x\in [-h_0,h_0]}$$$${u_0}(x)$$. A direct calculation gives $$v_1(t)=e^{-Lt}\min _{x\in [-h_0,h_0]} $$$${u_0}(x)<\delta $$ for $$t> 0$$. By the comparison principle, we obtain

$$\begin{aligned} u(t,x)\ge e^{-Lt}\min _{x\in [-h_0,h_0]}{u_0}(x) \quad \text{ for } t \in [0, T),\ x \in [g(t), h(t)]. \end{aligned}$$

This proves the first part of the lemma with $C_1(T)=e^{-LT}\min _{x\in [-h_0,h_0]}{u_0}(x)$.

Define

$$\begin{aligned} U(t):= \int _{g(t)}^{h(t)} u(t, x) \, dx. \end{aligned}$$

Then, for $$t \in (0, T)$$,

$$\begin{aligned}\begin{aligned} U'(t)&= h'(t)u(t, h(t)) - g'(t)u(t, g(t)) + \int _{g(t)}^{h(t)} u_t(t, x) \, dx\\&=\delta h'(t) - \delta g'(t) + \int _{g(t)}^{h(t)} \big [du_{xx} + f(t,x, u(t,x))\big ] \, dx\\&=\int _{g(t)}^{h(t)} f(t,x,u(t,x)) \, dx \ge -L \int _{g(t)}^{h(t)} u(t,x) \, dx = -L U(t). \end{aligned} \end{aligned}$$

Thus, $$U(t) \ge U(0)e^{-Lt} > 0 $$$$ \text { for } t \in (0, T)$$. Recalling that $$0<u(t, x) \le C_2 \text { for } $$$$0\le t<T, \ x\in [g(t), h(t)]$$, we deduce

$$\begin{aligned} C_2[h(t)-g(t)]\ge U(t) \ge U(0)e^{-Lt} \ge 2h_0 \min _{x\in [-h_0,h_{0}]} u_0(x)e^{-Lt}, \end{aligned}$$

which yields

$$\begin{aligned} h(t)-g(t)\ge \frac{2h_0}{C_2}e^{-Lt} \min _{x\in [-h_0,h_{0}]} u_0(x). \end{aligned}$$

This completes the proof of the second part. $\square$

The proof of the following a priori estimates require new techniques beyond (Du 2024).

#### Lemma 5.5

Assume (3.2) holds and (*u*, *g*, *h*) is a solution to (3.1) defined for $t \in [0, T)$ with $T \in (0, \infty )$. Then there exists a constant $C_4(T) > 0$ such that

$$\begin{aligned} |g'(t)|, |h'(t)|\le C_4(T) \quad \textrm{for }\ t \in [0, T). \end{aligned}$$

We will see from the proof below that $C_4(T)$ depends only on *T*, *d*, $\delta$, $u_0$ and *f*.

#### Proof

In the following, we only prove the boundedness of $|h'|$, as the proof for $|g'|$ is similar.

The key is to construct a lower solution to bound $h'(t)$ from below. Define, for some constants $0< c < 1$, $k > 0$ and $m > 0$ to be specified later,

$$\begin{aligned} {\left\{ \begin{array}{ll} \theta (s) := c(e^{-s/c} - 1) \text{ for } s \ge 0,\\ {\underline{u}}(t,x): = \delta [\theta ((h(t)-x)m) + 1] \text{ for } t \ge 0, \, x \in [h(t)-k/m, h(t)]. \end{array}\right. } \end{aligned}$$

We are going to show that, for suitable choices of (*c*, *k*, *m*),

5.18 $$\begin{aligned} {\left\{ \begin{array}{ll} {\underline{u}}_t< d {\underline{u}}_{xx} + f(t,x, {\underline{u}}), & t \in (0, T), \, x \in [h(t)-k/m, h(t)], \\ {\underline{u}}(t,h(t)-k/m) < u(t,h(t)-k/m), \ {\underline{u}}(t, h(t)) = \delta , & t \in [0, T), \\ {\underline{u}}(0, x) \le u_0(x), & x \in [h_0-k/m, h_0], \end{array}\right. } \end{aligned}$$

and

5.19 $$\begin{aligned} h'(t) \ge -\frac{d}{\delta } {\underline{u}}_x(t, h(t)) = -dm, \quad t \in [0, T). \end{aligned}$$

We first choose $c \in (0,1)$ close to 1. Then define

5.20 $$\begin{aligned} \begin{aligned}&k:= -c \ln \left( \frac{C_1}{c \delta } + 1 - \frac{1}{c}\right) > 0,\\&m:= \max \left\{ \frac{\max _{x \in [-h_0, h_0]} |u_0'(x)|}{\delta } e^{k/c}, \frac{2k}{C_3}, \sqrt{\frac{F_* c e^{k/c}}{d \delta (1-c)}}\right\} \end{aligned} \end{aligned}$$

with

5.21 $$\begin{aligned} F_* := \sup _{t\ge 0, x\in {\mathbb {R}}, u \in [0, C_2]} |f(t,x,u)|, \end{aligned}$$

where $C_1$, $C_2$, $C_3$ are as given in Lemma 5.4. Note that we always have $C_1 < \delta$.

From Lemma 5.4 and the expression for *k*, we obtain

$$\begin{aligned}&h(t) - k/m \in (g(t), h(t)) \quad \textrm{for}\ t \in [0, T), \\&{\underline{u}}(t, h(t) - k/m) = C_1 \le u(t, h(t) - k/m) \quad \textrm{for}\ t \in [0, T). \end{aligned}$$

The definition of ${\underline{u}}$ gives ${\underline{u}}(t, h(t)) = \delta$. Thus, the desired relations in the second line of (5.18) hold.

A direct calculation gives

$$\begin{aligned} {\underline{u}}_x(t,x) = m \delta e^{-[(h(t)-x)m]/c} \ge m \delta e^{-k/c} \quad \text{ for } t \ge 0, \, x \in [h(t)-k/m, h(t)]. \end{aligned}$$

This and (5.20) clearly yield ${\underline{u}}_x(0,x) \ge u_0'(x)$ for $x \in [h_0-k/m, h_0]$, which in turn implies, using ${\underline{u}}(0, h_0) = u_0(h_0) = \delta$, the following estimate

$$\begin{aligned} {\underline{u}}(0,x) \le u_0(x) \quad \text{ for } x \in [h_0-k/m, h_0), \end{aligned}$$

which is the third inequality in (5.18).

It remains to prove the first inequality in (5.18), and in doing so we will complete the proof of the lemma.

**Step 1:** We show that the first inequality in (5.18) holds for $t \in [0, T_1)$, where $T_1 \le T$ is given by

$$\begin{aligned} T_1 := \sup \{t\in [0, T) : h'(s) > -\frac{d}{\delta } {\underline{u}}_x(s, h) = -dm \ \mathrm{for\ all}\ s \in [0, t)\}. \end{aligned}$$

Since ${\underline{u}}_x(0, h_0) = m \delta > u_0'(h_0)$, by the continuity of $h'(t)$ and $u_x(t,h(t))$ for $t\in [0, T)$, we have

$$\begin{aligned} h'(0) = -\frac{d}{\delta } u_0'(h_0) > -\frac{d}{\delta } {\underline{u}}_x(0, h_0) = -dm, \end{aligned}$$

which implies that $T_1$ is well-defined, and

5.22 $$\begin{aligned} h'(s) \ge -\frac{d}{\delta } {\underline{u}}_x(s, h) = -dm \quad \mathrm{for\ all}\ s \in [0, T_1). \end{aligned}$$

(We will eventually show that $T_1$ equals *T*.)

A direct computation gives

$$\begin{aligned} {\underline{u}}_t = -h'(t) \delta m e^{-(h(t)-x)m/c}, \quad {\underline{u}}_x = \delta m e^{-(h(t)-x)m/c}, \quad {\underline{u}}_{xx} = \frac{\delta m^2}{c} e^{-(h(t)-x)m/c}. \end{aligned}$$

Using (5.20), (5.21) and (5.22), for $t \in (0, T_1), \, x \in [h(t)-k/m, h(t)]$, we have

$$\begin{aligned} {\underline{u}}_t&\le d \delta m^2 e^{-(h(t)-x)m/c} \le cd {\underline{u}}_{xx}=d{\underline{u}}_{xx}-d(1-c)\frac{\delta m^2}{c} e^{-(h(t)-x)m/c}\\&\le d{\underline{u}}_{xx}-d(1-c)\frac{\delta m^2}{c} e^{-k/c} \le d{\underline{u}}_{xx}- F_* \le d {\underline{u}}_{xx} + f(t,x,{\underline{u}}). \end{aligned}$$

Hence, the first inequality in (5.18) holds for $0<t< T_1$.

**Step 2.** We prove that for $t \in [0, T_1)$,

5.23 $$\begin{aligned} -dm \le h'(t) \le \frac{8}{3}M_*(C_2-\delta )d/\delta \end{aligned}$$

for some $M_*$ defined later.

The lower bound for $h'(t)$ has been proved in (5.22). So we only need to prove the upper bound for $h'(t)$. Define

$$\begin{aligned} \begin{aligned}&\Omega := \left\{ (t, x): 0 \le t< T_1, \, h(t) - 1/(2M_*)< x < h(t)\right\} , \\&{\bar{u}}(t, x):= \frac{4}{3} \left( C_2 - \delta \right) \left[ 2M_*(h(t)-x) - M_*^2(h(t)-x)^2\right] + \delta . \end{aligned} \end{aligned}$$

with

5.24 $$\begin{aligned} M_* := \max \left\{ \frac{1}{2C_3}, \sqrt{\frac{3F_*}{8d(C_2-\delta )}} + m, \frac{3\max _{x \in [-h_0, h_0]}|u_0'(x)|}{4(C_2-\delta )}\right\} , \end{aligned}$$

where *m* and $F_*$ are given by (5.20) and (5.21), respectively, $C_2$ and $C_3$ are given by Lemma 5.4. A direct calculation gives

$$\begin{aligned}&\Omega \subset \{(t, x) : 0 \le t< T_1, g(t)< x < h(t)\}, \\&\delta = {\bar{u}}(t, h(t)) \le {\bar{u}}(t, x) \le {\bar{u}}\left( t, h(t) - 1/(2M_*)\right) = C_2 \text { for } (t, x) \in \Omega , \\&{\bar{u}}\left( t, h(t) - 1/(2M_*)\right) = C_2 \ge u\left( t, h(t) - 1/(2M_*)\right) . \end{aligned}$$

Moreover, for $(t, x) \in \Omega$, by (5.22), the estimate ${\bar{u}}(t,x) \le C_2$, (5.21) and (5.24), we deduce

$$\begin{aligned} {\bar{u}}_t - d {\bar{u}}_{xx} - f(t,x,{\bar{u}})&=\frac{4}{3} \left( C_2 - \delta \right) h'(t) \left[ 2M_* - 2M_*^2(h(t)-x)\right] + d\frac{4}{3}\left( C_2 - \delta \right) 2M_*^2 - f(t,x,{\bar{u}}) \\&\ge -2dM_*m\frac{4}{3}(C_2 - \delta )[1 - M_*(h(t)-x)] + d\frac{4}{3}(C_2 - \delta ) 2M_*^2 - F_* \\&\ge -2dM_*m\frac{4}{3}(C_2 - \delta ) + d\frac{4}{3}(C_2 - \delta ) 2M_*^2 - F_* \\&= \frac{8}{3}dM_*[M_*-m](C_2 - \delta ) - F_* \ge 0. \end{aligned}$$

We show next that

$$\begin{aligned} {\bar{u}}(0, x) \ge u_0(x) \text { for } x \in \left[ h_0 - 1/(2M_*), h_0\right] . \end{aligned}$$

Indeed, by (5.24), for such *x*,

$$\begin{aligned} {\bar{u}}_x(0, x) = -\frac{8}{3}M_*(C_2 - \delta )[1 - M_*(h_0 - x)] \le -\frac{4}{3}M_*(C_2 - \delta ) \le u_0'(x). \end{aligned}$$

Thus, it follows from $${\bar{u}}(0, h_0)  $$$$ = u_0(h_0) = \delta $$ that $${\bar{u}}(0, x) $$$$ \ge u_0(x)$$ for $$x \in $$$$[h_0 - 1/(2M_*), h_0]$$.

The above analysis allows us to apply the usual comparison principle over

$$\begin{aligned} \left\{ (t, x): 0 \le t< T_1, \, h(t) - 1/(2M_*)< x < h(t)\right\} \end{aligned}$$

to conclude that $u(t, x) \le {\bar{u}}(t, x)$ in this region. Since $u(t, h(t)) = {\bar{u}}(t, h(t)) = \delta$, it follows that

$$\begin{aligned} u_x(t, h(t)) \ge {\bar{u}}_x(t, h(t)) = -\frac{8}{3}M_*(C_2 - \delta ) \quad \text {for } t \in [0, T_1). \end{aligned}$$

Hence,

$$\begin{aligned} -dm \le h'(t) = -\frac{d}{\delta } u_x(t, h(t)) \le \frac{8}{3}M_*(C_2 - \delta )\frac{d}{\delta } \quad \text{ for } t \in (0, T_1). \end{aligned}$$

**Step 3.** We prove that (5.19) holds, which then allows us to repeat the argument in Step 2 with $T_1$ replaced by *T* to conclude that (5.23) holds for all $t \in [0, T)$.

If $T_1 = T$, then the proof is complete. Suppose now $T_1 < T$, which implies

5.25 $$\begin{aligned} h'(T_1) = -\frac{d}{\delta } {\underline{u}}_x(T_1, h(T_1)) = -dm. \end{aligned}$$

Let $$w:= u - {\underline{u}}$$. Then

$$\begin{aligned} {\left\{ \begin{array}{ll} w_t> dw_{xx} + f(t,x,u) - f(t,x, {\underline{u}}) = dw_{xx} + c w, & t \in (0, T_1], \, x \in [h(t)-k/m, h(t)], \\ w(t, h(t)-k/m) > 0, \ w(t, h(t)) = 0, & t \in [0, T_1], \\ w(0, x) \ge 0, & x \in [h_0-k/m, h_0], \end{array}\right. } \end{aligned}$$

for some $c \in L^\infty$. By the comparison principle,

$$\begin{aligned} w(t, x) > 0 \quad \text{ for } t \in (0, T_1], \, x \in [h(t)-k/m, h(t)). \end{aligned}$$

Since $w(T_1, h(T_1))=0$, we may now apply the Hopf boundary lemma to conclude that $w_x(T_1, h(T_1)) <0$. Therefore $u_x(T_1, h(T_1)) < {\underline{u}}_x(T_1, h(T_1))$ and

$$\begin{aligned} h'(T_1) = -\frac{d}{\delta } u_x(T_1, h(T_1)) > -\frac{d}{\delta } {\underline{u}}_x(T_1, h(T_1)) = -dm, \end{aligned}$$

which contradicts (5.25). Hence, $T_1 = T$ always holds. This indicates (5.23) holds for $t \in [0, T)$. The proof is complete. $\square$

### 5.3 Proof of the global existence theorem

#### 5.4 Proof of Theorem 3.2

By Theorem 3.1, we may assume that (3.1) has a unique solution (*u*, *g*, *h*) defined on some maximal time interval $(0, T_m)$ with $T_m\in (0,\infty ]$, and

$$\begin{aligned} h, g \in C^{1+\frac{\beta }{2}}((0, T_m)), \quad u \in C^{1+\frac{\beta }{2}, 2+\beta }(\Omega _{T_m}), \end{aligned}$$

where $\Omega _{T_m}:= \{(t, x): t \in (0, T_m), x \in [g(t), h(t)]\}$. To complete the proof, we must demonstrate that $T_m = \infty$. Suppose $T_m < \infty$. Then, by the proof of Theorem 3.1, along with Lemmas 5.4 and 5.5, there exist positive constants $C_1, C_2=C_2(T_m)$, such that for $t \in [0, T_m)$ and $x \in [g(t), h(t)]$,

$$\begin{aligned} 0 \le u(x, t) \le C_1, \quad |h'(t)| + |g'(t)| \le C_2, \quad |h(t)|, |g(t)| \le C_2 t + h_0. \end{aligned}$$

For any small constant $\varepsilon > 0$, it follows from the proof of Theorem 3.1 and Lemmas 5.4 and 5.5 that $u \in C^{1+\beta , \frac{1+\beta }{2}}(\Omega _{T_m-\varepsilon })$. Thus, as in Step 4 of the proof of Theorem 3.1, applying Schauder’s estimates, for any fixed $0< T_0 < T_m - \varepsilon$, we obtain $\Vert u\Vert _{C^{2+\beta ,1+\frac{\beta }{2} }(\Omega _{T_m - \varepsilon } \setminus \Omega _{T_0})} \le Q^*$, where $Q^*$ depends on $T_0$, $T_m$, and $C_i$ for $i = 1, 2$, but is independent of $\varepsilon$. Since $\varepsilon > 0$ can be made arbitrarily small, it follows that for any $t \in [T_0, T_m)$,

$$\begin{aligned} \Vert u(t, \cdot )\Vert _{C^{2+\beta }([g(t), h(t)])} \le Q^*. \end{aligned}$$

By repeating the arguments used in the proof of Theorem 3.1, we can conclude that there exists $T > 0$ small, depending on $Q^*$ and $C_i$ $(i = 1, 2)$, such that the solution to (3.1) with initial time $T_m - \frac{T}{2}$ can be extended uniquely to $t=T_m-\frac{T}{2}+T>T_m$, a contradiction to the definition that $T_m$ is the maximal time interval for the solution. Thus, we must have $T_m = \infty$. $\square$

### 5.4 Proof of Theorem 3.3

Let us first note that $f(t,x,1)\equiv 0$ and $f(t,x,u)\le {{\bar{f}}}(u)<0$ for $u>1$ imply ${{\bar{f}}}(1)=0$. Let $M_0=\max \{\Vert u_0\Vert _\infty , 1\}$ and *v*(*t*) be the solution of

$$\begin{aligned} v'={{\bar{f}}}(v),\ v(0)=M_0. \end{aligned}$$

Since ${{\bar{f}}}(v)<0$ for $v>1$, we clearly have $1\le v(t)\le M_0$ and $v(t)\rightarrow 1$ as $t\rightarrow \infty$. Since $f(t,x,u)\le {{\bar{f}}}(u)$ for $u\ge 1$, we obtain

$$\begin{aligned} v_t-dv_{xx}={{\bar{f}}}(v)\ge f(t,x,v) \text{ for } t>0, x\in [g(t), h(t)]. \end{aligned}$$

We also have $v\ge 1>\delta =u$ for $x\in \{g(t), h(t)\}$ and $v(0)=M_0\ge u(0,x)$ for $x\in [-h_0, h_0]$. Therefore the standard comparison principle over the region $\{(t,x): t>0, x\in [g(t), h(t)]\}$ implies $u(t,x)\le v(t)$ in this region. It follows that

5.26 $$\begin{aligned} \limsup _{t\rightarrow \infty }u(t,x)\le \lim _{t\rightarrow \infty }v(t)=1 \text{ uniformly } \text{ for } x\in [g(t), h(t)]. \end{aligned}$$

To bound *u*(*t*, *x*) from below, we first make use of (3.4) to show that there exists $T_0>0$ such that

5.27 $$\begin{aligned} u(t, x) \ge \delta \text{ for } t\ge T_0 \text{ and } x\in [g(t), h(t)]. \end{aligned}$$

Similar to the proof of Theorem 1.1, since ${\underline{f}}$ satisfies $\mathbf{(f_A)}$, we are able to choose a function ${\hat{f}} \in C^1$ sufficiently close to ${\underline{f}}$ in $L^\infty$ such that ${\hat{f}}(s) \le {\underline{f}}(s)$ for $s \ge 0$, and ${{\hat{f}}}$ satisfies $\mathbf {(F_b)}$ with $(P, Q)=({\hat{\theta }}, \delta )$ for some ${\hat{\theta \in }} [\theta , \delta )\cap (0,\delta )$. Then, by Lemma 2.1, the traveling wave problem (2.4) has a solution pair $(c,q)=(c_0,q_0)$ with $c_0>0$ and $q_0(\cdot )$ strictly increasing.

The same reasoning as in the proof of Theorem 1.1 shows that for some $L > 0$ sufficiently large,

$$\begin{aligned} {\underline{u}}(t, x):= \max \{q_0(ct - x - L), q_0(ct + x - L)\} \end{aligned}$$

satisfies (in the weak sense)

$$\begin{aligned} {\underline{u}}_t \le d {\underline{u}}_{xx} + {\underline{f}}({\underline{u}})\le f(t,x,{\underline{u}}) \quad \text {for } t > 0, \; x \in {\mathbb {R}}. \end{aligned}$$

Additionally,

$$\begin{aligned} 0 \le {\underline{u}}(t, x) \le \delta \le u(t,x) \text{ for } t>0,\ x\in \{g(t), h(t)\},\end{aligned}$$

and

$$\begin{aligned} {\underline{u}}(0, x) \le u_0(x) \quad \text {for } x \in [-h_0, h_0]. \end{aligned}$$

Therefore we can apply the standard comparison principle over $\{(t, x): t > 0, x \in [g(t), h(t)]\}$ to deduce $u(t, x) \ge {\underline{u}}(t, x)$ in this region.

Moreover, using

$$\begin{aligned} \lim _{t \rightarrow \infty } \Vert {\underline{u}}(t,\cdot ) - \delta \Vert _{L^\infty ({\mathbb {R}})} = 0, \end{aligned}$$

and $\delta > \theta$, there exists $t_0 > 0$ such that

$$\begin{aligned} {u}(t, x) \ge {\underline{u}}(t, x) >\theta \quad \text{ for } t \ge t_0, \; x \in [g(t), h(t)]. \end{aligned}$$

As in the proof of Theorem 1.1, let $m_0:= \min _{x \in [g(t_0), h(t_0)]} u(t_0, x)$. Then $\delta \ge m_0 >\theta$. Consider the auxiliary ODE problem:

$$\begin{aligned} w' = {\underline{f}}(w), \quad w(t_0) = m_0. \end{aligned}$$

Since ${\underline{f}}(u)> 0$ in $(\theta , 1)$, the solution *w*(*t*) is increasing in *t*, and $w(t) \rightarrow 1$ as $t \rightarrow \infty$. Thus, there exists $T_0 \ge t_0$ such that $w(T_0) = \delta$ and $m_0 \le w(t) \le \delta$ for $t \in [t_0, T_0]$. By the comparison principle over the region $\{(t, x): t_0 \le t \le T_0, \; g(t) \le x \le h(t)\}$, we obtain $u(t, x) \ge w(t)$ in this region. In particular,

$$\begin{aligned} u(T_0, x) \ge w(T_0) = \delta \quad \text{ for } x \in [g(T_0), h(T_0)]. \end{aligned}$$

Comparing *u*(*t*, *x*) with the constant function ${\underline{u}}_1(t, x) \equiv \delta$ over $\{(t, x): T_0 \le t < \infty , \; g(t) \le x \le h(t)\}$, we conclude by the usual comparison principle that $u(t, x) \ge \delta$ for $x \in [g(t), h(t)]$ and $t \in [T_0, \infty )$. This proves (5.27),

Next using $T_0$ in (5.27) as the initial time and consider the problem

5.28 $$\begin{aligned} {\left\{ \begin{array}{ll} {\tilde{u}}_t-d {\tilde{u}}_{xx}={\underline{f}}( {\tilde{u}}), & t>T_0,\; {\tilde{g}}(t)<x< {\tilde{h}}(t),\\ {\tilde{u}}(t, {\tilde{g}}(t))={\tilde{u}}(t,{\tilde{h}}(t))=\delta , & t>T_0,\\ {\tilde{g}}'(t)=-\frac{d}{\delta } {\tilde{u}}_{x}(t, {\tilde{g}}(t)),& t>T_0,\\ {\tilde{h}}'(t)=-\frac{d}{\delta }{\tilde{u}}_x(t, {\tilde{h}}(t)), & t>T_0,\\ {\tilde{g}}(T_0)=g(T_0)+\epsilon ,\ {\tilde{h}}(T_0)=h(T_0)-\epsilon , \ {\tilde{u}}(T_0,x)=\delta , & g(T_0)+\epsilon \le x\le h(T_0)-\epsilon , \end{array}\right. } \end{aligned}$$

where $\epsilon >0$ is small.

Since ${\underline{f}}$ satisfies $\mathbf{(f_A)}$ and $\delta >\theta ^*_{{\underline{f}}}$, Theorem 1.1 applies to (5.28). With (5.27) holding for all $t\ge T_0$, and $f(t,x,u)\ge {\underline{f}}(u)$, we see that (*u*, *g*, *h*) is an upper solution to (5.28) and can apply Lemma 5.2 to conclude that

$$\begin{aligned} {[}{\tilde{g}}(t), {\tilde{h}}(t)]\subset (g(t), h(t)),\ \ {\tilde{u}}(t,x)\le u(t,x) \text{ for } t\ge T_0, \ x\in [{\tilde{g}}(t), {\tilde{h}}(t)]. \end{aligned}$$

The conclusions of the theorem now follow directly from this, (5.26) and the results in Theorem 1.1 for $({\tilde{u}}, {\tilde{g}}, {\tilde{h}})$. $\square$

### 5.5 Proof of Theorem 3.4

We complete the proof by three steps.

**Step 1.** We show that there exists $T_0>0$ such that $u(t,x)\le \delta$ for $t\ge T_0$ and $x\in [g(t), h(t)]$.

Let $m_0=\Vert u_0\Vert _\infty +1$ and consider the ODE problem

$$\begin{aligned} w'={{\bar{f}}}(w),\ w(0)=m_0. \end{aligned}$$

Since ${{\bar{f}}}(1)=0$, ${{\bar{f}}}(u)<0$ for $u>1$ and $m_0\ge 1+\delta$, the unique solution *w*(*t*) is strictly decreasing and $w(t)\rightarrow 1$ as $t\rightarrow \infty$. Therefore there exists a unique $T_0>0$ such that $w(T_0)=\delta \in (1, m_0)$. We may now compare *u*(*t*, *x*) with *w*(*t*) in the region $\{(t,x): t\in [0, T_0], \ x\in [g(t), h(t)]\}$ by the usual comparison principle, and conclude that $u(t,x)\le w(t)$ in this region. It follows that $u(T_0, x)\le \delta$ for $x\in [g(T_0, h(T_0)]$. Since $f(t,x,\delta )\le {{\bar{f}}}(\delta )<0$, we see that over the region $\{(t,x): t\ge T_0, \ x\in [g(t), h(t)]\}$, ${{\bar{u}}}\equiv \delta$ is always an upper solution to the initial boundary value problem satisfied by *u*(*t*, *x*), and therefore $u(t,x)\le \delta$ in this region.

**Step 2.** We show that there exists $\xi ^*\in {\mathbb {R}}$ such that

5.29 $$\begin{aligned} \lim _{t\rightarrow \infty } g(t)=\lim _{t\rightarrow \infty } h(t)=\xi ^*. \end{aligned}$$

The conclusion proved in Step 1 above implies that for $t\ge T_0$,

$$\begin{aligned} h'(t)=-\frac{d}{\delta }u_x(t, h(t))\le 0,\ g'(t)=-\frac{d}{\delta }u_x(t, g(t))\ge 0. \end{aligned}$$

Therefore

$$\begin{aligned} h_\infty :=\lim _{t\rightarrow \infty } h(t) \text{ and } g_\infty :=\lim _{t\rightarrow \infty } g(t) \text{ exist, } \text{ and } [g_\infty , h_\infty ]\subset [g(T_0, h(T_0)]. \end{aligned}$$

It remains to show $g_\infty =h_\infty$.

Arguing indirectly we assume $h_\infty >g_\infty$ and seek a contradiction. Define

$$\begin{aligned} y=\frac{[h(t)-x] g_\infty +[x-g(t)]h_\infty }{h(t)-g(t)},\ \ U(t,y)=u(t,x). \end{aligned}$$

By direct calculation we see that *U* satisfies

5.30 $$\begin{aligned} {\left\{ \begin{array}{ll} U_t-d\Big [\frac{h_\infty -g_\infty }{h(t)-g(t)}\Big ]^2U_{yy}+\frac{h'(t)g_\infty -g'(t)h_\infty -[h'(t)-g'(t)]y}{h(t)-g(t)}U_y=F(t,y,U), & t>0,\ y\in {[}g_\infty , h_\infty ],\\ U(t, g_\infty )=U(t, h_\infty )=\delta , & t>0,\\ h'(t)=-\frac{d}{\delta }\frac{h_\infty -g_\infty }{h(t)-g(t)}U_y(t, h_\infty ), & t>0,\\ g'(t)=-\frac{d}{\delta }\frac{h_\infty -g_\infty }{h(t)-g(t)}U_y(t, g_\infty ), & t>0,\\ U(0, y)=u_0\Big (\frac{2h_0y-(g_\infty +h_\infty )h_0}{h_\infty -g_\infty }\Big ), & y\in [g_\infty , h_\infty ], \end{array}\right. } \end{aligned}$$

where

$$\begin{aligned} F(t,y,U):=f(t, \frac{h(t)(y-g_\infty )+g(t)(h_\infty -y)}{h_\infty -g_\infty }, U). \end{aligned}$$

We easily see that $$\Vert F(\cdot , \cdot , U(\cdot ,\cdot )) $$$$\Vert _{L^\infty ([0,\infty )\times [g_\infty , h_\infty ])}<\infty $$. Since $$h'(t)\ge 0\ge g'(t)$$ for $$t\ge T_0$$, from Step 2 in the proof of Lemma 5.5 we see that $$\Vert h'\Vert _{L^{\infty }([0,\infty ))}+\Vert g'\Vert _{L^\infty ([0,\infty ))}<\infty $$. Therefore, in the first line of (5.30), the coefficient of $$U_{yy}$$ is uniformly continuous, and the coefficient of $$U_y$$ belongs to $$L^\infty ([0,\infty )\times [g_\infty , h_\infty ])$$. We may now apply standard $$L^p$$ theory to the initial boundary value problem consisting of the first, second and fifth lines of (5.30) to conclude that, for any $$p>1$$,

$$\begin{aligned} \Vert U\Vert _{W^{1,2}_p([n, n+2]\times [g_\infty , h_\infty ])}\le C_p \end{aligned}$$

for all integers $n\ge 1$ and some $C_p>0$ independent of *n*. Taking *p* sufficiently large and use the Sobolev embedding theorem, we see from the third and fourth equations in (5.30) that $h'(t)$ and $g'(t)$ are uniformly continuous in *t* for $t\ge 1$. This implies that $h'(t)\rightarrow 0$ and $g'(t)\rightarrow 0$ as $t\rightarrow \infty$, since otherwise, say there exists $s_n$ increasing to $\infty$ as $n\rightarrow \infty$ such that $h'(s_n)\ge \sigma >0$ for all *n*, then we can find $\epsilon >0$ small so that $h'(s)\ge \sigma /2$ for $s\in [s_n-\epsilon , s_n+\epsilon ]$ and all $n\ge 1$, which together with $h'(t)\ge 0$ for $t>T_0$ implies $h_\infty =\infty$.

Let $\{t_n\}$ be an arbitrary sequence increasing to $\infty$ as $n\rightarrow \infty$, and define

$$\begin{aligned} U_n(t,y):=U(t_n+t, y). \end{aligned}$$

Then applying the $L^p$ estimates to the initial boundary value problem satisfied by $U_n$ (which is a simple variation of the first, second and fifth lines of (5.30)) and using the Sobolev embedding theorem, we see that, for any $\alpha \in [\beta ,1)$, subject to a subsequence, $U_n\rightarrow {\tilde{U}}$ in $C_{loc}^{(1+\alpha )/2, 1+\alpha }({\mathbb {R}}\times [g_\infty , h_\infty ])$. By Step 1 we may assume $U_n\le \delta$ for all $n\ge 1$ and hence ${\tilde{U}}\le \delta$.

We claim that ${\tilde{U}}$ is a classical solution of

5.31 $$\begin{aligned} {\left\{ \begin{array}{ll} {\tilde{U}}_t-d{\tilde{U}}_{yy}={\tilde{F}}(t, y, {\tilde{U}}), & t\in {\mathbb {R}},\ y\in [g_\infty , h_\infty ],\\ {\tilde{U}}(t, g_\infty )={\tilde{U}}(t, h_\infty )=\delta , & t\in {\mathbb {R}},\\ {\tilde{U}}_y(t, g_\infty )={\tilde{U}}_y(t, h_\infty )=0, & t\in {\mathbb {R}}, \end{array}\right. } \end{aligned}$$

where ${\tilde{F}}(t,y,\xi )$ is a function in $C^{\beta /2, \beta , 1-0}({\mathbb {R}}\times [g_\infty , h_\infty ]\times [0, \delta ])$, with $\xi \rightarrow {\tilde{F}}(t,y,\xi )$ Lipschitz continuous over $[0, \delta ]$ uniformly for $t\in {\mathbb {R}}$ and $y\in [g_\infty , h_\infty ]$, and ${\tilde{F}}(t,y, \xi )\le {{\bar{f}}}(\xi )<0$ for $\xi \in (1, \delta ]$ and all $t\in {\mathbb {R}},\ y\in [g_\infty , h_\infty ]$.

Indeed, by our assumptions on *f*(*t*, *x*, *u*) we know that, for any $T>0$, there exists a positive integer $n_T$ such that

$$\begin{aligned} {{\hat{F}}}_n(t, y, \xi ):=F(t_n+t, y,\xi ), \ n\ge n_T \end{aligned}$$

form a sequence in $C([-T, T]\times [g_\infty , h_\infty ]\times [0, \delta ])$ which is uniformly bounded and equicontinuous. Therefore by passing to a subsequence, ${{\hat{F}}}_n(t,y,\xi )\rightarrow {\tilde{F}}_T(t,y,\xi )$ as $n\rightarrow \infty$ in the space $C([-T, T]\times [g_\infty , h_\infty ]\times [0, \delta ])$. Taking a sequence $T_k\rightarrow \infty$ as $k\rightarrow \infty$ and using a standard diagonal process of selecting subsequences, we can find a subsequence of $\{t_n\}$, still denoted by $\{t_n\}$ for convenience of notation, and a function ${\tilde{F}}\in C({\mathbb {R}}\times [g_\infty , h_\infty ]\times [0, \delta ])$ such that, as $n\rightarrow \infty$,

$$\begin{aligned}{\hat{F}}_n(t, y, \xi )\rightarrow {\tilde{F}}(t,y, \xi ) \text{ uniformly } \text{ in } [-T, T]\times [g_\infty , h_\infty ]\times [0, \delta ] \text{ for } \text{ every } T>0\text{. } \end{aligned}$$

Since

$$\begin{aligned} U_n(t,y)\rightarrow {\tilde{U}}(t, y) \text{ and } \ Y_n(t,y):=\frac{h(t_n+t)(y-g_\infty )+g(t_n+t)(h_\infty -y)}{h_\infty -g_\infty }\rightarrow y \end{aligned}$$

uniformly in $[-T, T]\times [g_\infty , h_\infty ]$ as $n\rightarrow \infty$, we see that for any $T>0$,

$$\begin{aligned} \begin{aligned} 0&\le |{{\hat{F}}}_n(t, Y_n(t,y), U_n(t,y))-{\tilde{F}}(t,y, {\tilde{U}}(t,y))|\\&\le |{{\hat{F}}}_n(t, Y_n(t,y), U_n(t,y))-{\tilde{F}}(t,Y_n(t,y), U_n(t,y))|\\&\ \ \ \ \ \ + |{\tilde{F}}(t, Y_n(t,y), U_n(t,y))-{\tilde{F}}(t,y, {\tilde{U}}(t,y))|\\&\rightarrow 0 \text{ as } n\rightarrow \infty \text{ uniformly } \text{ in } [-T, T]\times [g_\infty , h_\infty ]\times [0, \delta ]. \end{aligned} \end{aligned}$$

One can now easily see from the above fact for ${{\hat{F}}}_n(t, Y_n(t,y), U_n(t,y))$ and the equations satisfied by $U_n(t,y)$ that for any $p>1$, ${\tilde{U}}$ is a $W^{1,2}_{p, loc}({\mathbb {R}}\times [g_\infty , h_\infty ])$ solution of (5.31).

Since $\xi \rightarrow f(t,x,\xi )$ is Lipschitz for $\xi \in [0, \delta ]$ uniformly for $t\ge 0$ and $x\in {\mathbb {R}}$, there exists a constant $L>0$ such that

$$\begin{aligned} |{{\hat{F}}}_n(t,y,\xi _1)-{{\hat{F}}}_n(t,y,\xi _2)|\le L|\xi _1-\xi _2| \text{ for } t\ge -t_n, y\in [g_\infty , h_\infty ],\ \xi _1,\xi _2\in [0, \delta ]. \end{aligned}$$

Letting $n\rightarrow \infty$ we deduce

$$\begin{aligned} |{\tilde{F}}(t,y,\xi _1)-{\tilde{F}}(t,y,\xi _2)|\le L|\xi _1-\xi _2| \text{ for } t\in {\mathbb {R}}, y\in [g_\infty , h_\infty ],\ \xi _1,\xi _2\in [0, \delta ]. \end{aligned}$$

Similarly, we can show ${\tilde{F}}(t,y,\xi )$ is $C^{\beta /2, \beta }$ in (*t*, *y*) for $t\in {\mathbb {R}}, y\in [g_\infty , h_\infty ],\xi \in [0,\delta ]$. Moreover, from $f(t,x,u)\le {{\bar{f}}}(u)$ we deduce

$$\begin{aligned} {{\hat{F}}}_n(t,y,\xi )\le {{\bar{f}}}(\xi ) \text{ for } t\ge -t_n, y\in [g_\infty , h_\infty ],\ \xi \in [1, \delta ]. \end{aligned}$$

Letting $n\rightarrow \infty$ we obtain ${\tilde{F}}(t,y,\xi )\le {{\bar{f}}}(\xi )$ for $t\in {\mathbb {R}},\ y\in [g_\infty , h_\infty ],\ \xi \in [1, \delta ]$. Thus ${\tilde{F}}$ has all the claimed properties.

With these properties of ${\tilde{F}}$, we may now use standard regularity iteration to see that ${\tilde{U}}$ is a classical solution of (5.31). Recall that Step 1 implies ${\tilde{U}}(t,y)\le \delta$, and ${\tilde{F}}(t,y,\delta )\le {{\bar{f}}}(\delta )<0$ further implies that we can use the usual strong maximum principle to conclude that ${\tilde{U}} (t, y)<\delta$ for $t\in {\mathbb {R}}$ and $y\in (g_\infty , h_\infty )$. Then the Hopf boundary lemma applied to the function $W(t,y):=\delta -{\tilde{U}}(t,y)$ at the boundary points $(t, g_\infty )$ and $(t, h_\infty )$ leads to ${\tilde{U}}_y(t, g_\infty )<0<{\tilde{U}}_y(t, h_\infty )$, which contradicts the third equation in (5.31). This contradiction proves the desired fact that $g_\infty =h_\infty$, and Step 2 is now finished.

**Step 3.** We prove that $u(t,x)\rightarrow \delta$ as $t\rightarrow \infty$ uniformly for $x\in [g(t), h(t)]$.

Since by Step 1, $u(t,x)\le \delta$ for $t\ge T_0$ and $x\in [g(t), h(t)]$, we only need to bound *u*(*t*, *x*) from below. We do this by using suitable lower solutions. By a translation of *x* we may assume that $\lim _{t\rightarrow \infty }g(t)=\lim _{t\rightarrow \infty } h(t)=0$. By the assumptions on *f*(*t*, *x*, *u*) we can find $M>0$ such that

$$\begin{aligned} f(t,x,\xi )\ge -M\xi \text{ for } \text{ all } t\ge 0, x\in {\mathbb {R}} \text{ and } \xi \in [0, \delta ]. \end{aligned}$$

For any small $\epsilon >0$, we consider the auxiliary problem

5.32 $$\begin{aligned} {\left\{ \begin{array}{ll} v_t-dv_{xx}=-Mv, & t>0, x\in [-\epsilon ,\epsilon ],\\ v(t,\pm \epsilon )=\delta , & t>0,\\ v(0, x)=0, & x\in [-\epsilon ,\epsilon ]. \end{array}\right. } \end{aligned}$$

Since 0 is a lower solution of the stationary problem of (5.32), the unique solution *v*(*t*, *x*) is increasing in *t*. As $\delta$ is an upper solution of (5.32), we further have $v(t,x)\le \delta$ for all $t>0$ and $x\in [-\epsilon ,\epsilon ]$. Therefore $v_*(x):=\lim _{t\rightarrow \infty } v(t,x)$ exists, and by standard regularity considerations $v_*$ is a stationary solution of (5.32), and $0<v_*(x)\le \delta$. The maximum principle indicates that $v_*$ is the unique stationary solution of (5.32). Define

$$\begin{aligned} v_\epsilon (x):=v_*(\epsilon x) \text{ for } x\in [-1,1]. \end{aligned}$$

Clearly

$$\begin{aligned} -d v_\epsilon ''=-\epsilon ^2 M v_\epsilon \text{ in } (-1, 1),\ v_\epsilon (\pm 1)=\delta . \end{aligned}$$

Since $\epsilon ^2Mv_\epsilon \rightarrow 0$ in $L^\infty ([-1,1])$ as $\epsilon \rightarrow 0$, it follows that $v_\epsilon \rightarrow v_0$ in $W^{2,p}([-1,1])$ for any $p>1$, and $v_0$ solves

$$\begin{aligned} -dv_0''=0,\ v_0(\pm 1)=\delta . \end{aligned}$$

Hence $v_0\equiv \delta$ and $v_\epsilon (x)\rightarrow \delta$ as $\epsilon \rightarrow 0$ uniformly for $x\in [-1,1]$.

Let $$T_\epsilon >T_0$$ satisfy

$$\begin{aligned} {[}g(t), h(t)]\subset (-\epsilon , \epsilon ) \text{ for } t\ge T_\epsilon . \end{aligned}$$

Then we can compare *u*(*t*, *x*) with $$v$$$$(t-T_\epsilon , x)$$ over the region $$\{(t,x): t\ge $$$$T_\epsilon , x\in [g(t), h(t)]\}$$ by the usual comparison principle to conclude that $$ u(t,x)\ge $$$$ v(t-T_\epsilon ,x)$$ in this region. It follows that

$$\begin{aligned} \liminf _{t\rightarrow \infty } u(t,x)\ge \lim _{t\rightarrow \infty } v(t-T_\epsilon ,x)=v_*(x)=v_\epsilon (x/\epsilon ) \text{ uniformly } \text{ for } x\in [g(t), h(t)]. \end{aligned}$$

Letting $\epsilon \rightarrow 0$ and using $v_0\equiv \delta$ we deduce

$$\begin{aligned} \liminf _{t\rightarrow \infty } u(t,x)\ge \delta \text{ uniformly } \text{ for } x\in [g(t), h(t)]. \end{aligned}$$

This completes Step 3, and the proof of Theorem 3.4 is finished. $\Box$

### 5.6 Proof of Conjecture 4 part (i) and Theorem 3.5

The proof of these two results share several ideas and techniques.

#### 5.6.1 Proof of Conjecture 4 part (i)

The case $\delta =\theta$ is simpler and will be proved after the case $\delta \in (0, \theta )$. The proof of this latter case consists of the following three steps.

**Step 1.** We prove that there exists $M>0$ such that $[g(t), h(t)]\subset (-M, M)$ for all $t>0$ and $\limsup _{t\rightarrow \infty } u(t,x)\le \delta$ uniformly for $x\in [g(t), h(t)]$.

Clearly ${\tilde{f}}(u):=f(u+\delta )$ is a combustion type function with ignition temperature ${{\tilde{\theta }}}=\theta -\delta$ and ${\tilde{f}}(1-\delta )=0$, ${\tilde{f}}(u)<0$ for $u>1-\delta$. Since $u_0(x)\le \theta$ for $x\in [-h_0, h_0]$ we can find ${{\bar{u}}}_0(x)\in X(h_0+1)$ such that

$$\begin{aligned} \theta \ge {{\bar{u}}}_0(x)\ge u_0(x) \text{ for } x\in [-h_0, h_0],\ {{\bar{u}}}_0(x)>\delta \text{ for } x\in (-h_0, h_0). \end{aligned}$$

We now consider the auxiliary free boundary problem

5.33 $$\begin{aligned} {\left\{ \begin{array}{ll} {\tilde{u}}_t-d{\tilde{u}}_{xx}={\tilde{f}}(u), & t>0, \ {\tilde{g}}(t)<x<{\tilde{h}}(t),\\ {\tilde{u}}(t,{\tilde{g}}(t))= {\tilde{u}}(t,{\tilde{h}}(t))=0,& t>0,\\ {\tilde{g}}'(t)=-\frac{d}{\delta }{\tilde{u}}_x(t, {\tilde{g}}(t)), & t>0,\\ {\tilde{h}}'(t)=-\frac{d}{\delta }{\tilde{u}}_x(t, {\tilde{h}}(t)),& t>0,\\ {\tilde{u}}(0,x)={{\bar{u}}}_0(x)-\delta ,\ -{\tilde{g}}(0)={\tilde{h}}(0)=h_0+1,& {\tilde{g}}(0)\le x\le {\tilde{h}}(0). \end{array}\right. } \end{aligned}$$

By Du and Lou (2015), there are three possibilities for the unique solution $({\tilde{u}}, {\tilde{g}}, {\tilde{h}})$ of (5.33) as $t\rightarrow \infty$: (i) spreading, (ii) vanishing and (iii) transition. Since ${{\bar{u}}}_0(x)-\delta \le {{\tilde{\theta }}}=\theta -\delta$, it follows from the comparison principle that ${\tilde{u}}(t,x)<{{\tilde{\theta }}}$ for $t>0$ and $x\in [{\tilde{g}}(t), {\tilde{h}}(t)]$. Therefore ${\tilde{f}}({\tilde{u}}(t,x))\equiv 0$, which implies, by the comparison principle again, ${\tilde{u}}(t,x)\le \max _{x\in [{\tilde{g}}(t_0), {\tilde{h}}(t_0)]}{\tilde{u}}(t_0,x)<{{\tilde{\theta }}}$ for $t>t_0>0$. Hence only vanishing is possible for $({\tilde{u}}, {\tilde{g}},{\tilde{h}})$. It follows that

$$\begin{aligned} \lim _{t\rightarrow \infty }[{\tilde{g}}(t), {\tilde{h}}(t)]=[{\tilde{g}}_\infty , {\tilde{h}}_\infty ] \text{ is } \text{ a } \text{ finite } \text{ interval, } [{\tilde{g}}(t), {\tilde{h}}(t)]\subset ({\tilde{g}}_\infty ,{\tilde{h}}_\infty ) \text{ for } t>0, \end{aligned}$$

and

$$\begin{aligned} {\tilde{u}}(t,x)\rightarrow 0 \text{ as } t\rightarrow \infty \text{ uniformly } \text{ for } x\in [{\tilde{g}}(t),{\tilde{h}}(t)]. \end{aligned}$$

On the other hand, the standard comparison principle implies $u(t,x)\le \theta$ for $t\ge 0$ and $x\in [g(t), h(t)]$. So $f(u(t,x))\equiv 0$ and it is easy to check that (*u*, *g*, *h*) and $({\tilde{u}}+\delta , {\tilde{g}}, {\tilde{h}})$ form a pair of upper and lower solutions as described in Lemma 5.2, which allows us to conclude that

$$\begin{aligned} {[}g(t), h(t)]\subset ({\tilde{g}}(t), {\tilde{h}}(t))\subset ({\tilde{g}}_\infty , {\tilde{h}}_\infty ),\ u(t,x)\le {\tilde{u}}(t,x)+\delta \text{ for } t>0,\ x\in [g(t), h(t)]. \end{aligned}$$

The desired conclusions clearly follow directly from the above estimates.

**Step 2.** We show that $u(t,x)\rightarrow \delta$ as $t\rightarrow \infty$ uniformly for $x\in [g(t), h(t)]$.

Let $M>0$ satisfy $[g(t), h(t)]\subset (-M, M)$ for $t>0$ as stated in Step 1. Then consider the auxiliary initial boundary value problem

$$\begin{aligned} {\left\{ \begin{array}{ll} v_t-d v_{xx}=0,& t>0,\ -M<x<M,\\ v(t, \pm M)=\delta ,& t>0,\\ v(0, x)=0, & x\in [-M, M]. \end{array}\right. } \end{aligned}$$

One easily sees that *v*(*t*, *x*) is increasing in *t* and $v(t,x)\rightarrow \delta$ as $t\rightarrow \infty$ uniformly for $x\in [-M, M]$. Moreover, we can compare *u*(*t*, *x*) with *v*(*t*, *x*) over the region $\{(t,x): t>0, x\in [g(t), h(t)]\}$ and use the usual comparison principle to conclude that $u(t,x)\ge v(t,x)$ in this region. It follows that

$$\begin{aligned} \liminf _{t\rightarrow \infty }u(t,x)\ge \lim _{t\rightarrow \infty } v(t,x)=\delta \text{ uniformly } \text{ for } x\in [g(t), h(t)]. \end{aligned}$$

This and the second conclusion in Step 1 imply that $u(t,x)\rightarrow \delta$ as $t\rightarrow \infty$ uniformly for $x\in [g(t), h(t)]$.

**Step 3.** We prove that $g_\infty :=\lim _{t\rightarrow \infty }g(t)$ and $h_\infty :=\lim _{t\rightarrow \infty }h(t)$ exist, and

$$\begin{aligned} \int _{-h_0}^{h_0}u_0(x)dx=\delta (h_\infty -g_\infty ). \end{aligned}$$

By Step 2, for any given small $\epsilon >0$, there exists $T=T_\epsilon >0$ such that

$$\begin{aligned} u(t,x)\in [\delta -\epsilon , \delta +\epsilon ]\subset (0, \theta ) \text{ for } t\ge T,\ x\in [g(t), h(t)]. \end{aligned}$$

Now we consider the following auxiliary problem

5.34 $$\begin{aligned} {\left\{ \begin{array}{ll} {{\hat{u}}}_t-d{{\hat{u}}}_{xx}=0, & t>0, \ {{\hat{g}}}(t)<x<{{\hat{h}}}(t),\\ {{\hat{u}}}(t,{{\hat{g}}}(t))= {{\hat{u}}}(t,{{\hat{h}}}(t))=\delta ,& t>0,\\ {{\hat{g}}}'(t)=-\frac{d}{\delta }{{\hat{u}}}_x(t, {{\hat{g}}}(t)), & t>0,\\ {\hat{h}}'(t)=-\frac{d}{\delta }{{\hat{u}}}_x(t, {{\hat{h}}}(t)),& t>0,\\ {\hat{u}}(0,x)={{\hat{u}}}_0(x),\ {{\hat{g}}}(0)=g(T)-\epsilon , {{\hat{h}}}(0)=h(T)+\epsilon ,& {{\hat{g}}}(0)\le x\le {{\hat{h}}}(0), \end{array}\right. } \end{aligned}$$

where ${{\hat{u}}}_0$ is a $C^2$ function satisfying

$$\begin{aligned} {{\hat{u}}}_0({{\hat{g}}}(0))={{\hat{u}}}_0({{\hat{h}}}(0))=\delta ,\ \max \{\delta , u(T, x)\} \le {{\hat{u}}}_0(x)\le \delta +\epsilon \text{ for } x\in [{{\hat{g}}}(0), {{\hat{h}}}(0)]. \end{aligned}$$

We easily see that the unique solution $({{\hat{u}}}, {{\hat{g}}}, {{\hat{h}}})$ of (5.34) satisfies $\delta +\epsilon \ge {{\hat{u}}}(t,x)\ge \delta$ for all $t>0$ and $x\in [{{\hat{g}}}(t), {{\hat{h}}}(t)]$. It follows that ${{\hat{h}}}'(t)\ge 0\ge {{\hat{g}}}'(t)$ for $t>0$. Define

$$\begin{aligned} {{\hat{U}}}(t):=\int _{{{\hat{g}}}(t)}^{{{\hat{h}}}(t)} {{\hat{u}}}(t,x)dx. \end{aligned}$$

Then, for $$t >0$$,

$$\begin{aligned}\begin{aligned} {{\hat{U}}}'(t)&= {{\hat{h}}}'(t){{\hat{u}}}(t, {{\hat{h}}}(t)) - {{\hat{g}}}'(t){{\hat{u}}}(t, {{\hat{g}}}(t)) + \int _{{{\hat{g}}}(t)}^{{{\hat{h}}}(t)} {{\hat{u}}}_t(t, x) \, dx\\&=\delta {{\hat{h}}}'(t) - \delta {{\hat{g}}}'(t) + \int _{{{\hat{g}}}(t)}^{{{\hat{h}}}(t)} d {{\hat{u}}}_{xx} \, dx\\&=\delta {{\hat{h}}}'(t) - \delta {{\hat{g}}}'(t) +d{{\hat{u}}}_x(t, {{\hat{h}}}(t))-d{{\hat{u}}}_x(t, {{\hat{g}}}(t))=0. \end{aligned} \end{aligned}$$

Hence ${{\hat{U}}}(t)={{\hat{U}}}(0)$ for $t>0$. It follows that, for all $t>0$,

$$\begin{aligned}{\left\{ \begin{array}{ll} {[}{{\hat{h}}}(t)-{{\hat{g}}}(t)](\delta +\epsilon )\ge {{\hat{U}}}(t)={{\hat{U}}}(0)\ge [{{\hat{h}}}(0)-{{\hat{g}}}(0)]\delta ,\\ {[}{{\hat{h}}}(t)-{{\hat{g}}}(t)]\delta \le {{\hat{U}}}(t)={{\hat{U}}}(0)\le [{{\hat{h}}}(0)-{{\hat{g}}}(0)](\delta +\epsilon ). \end{array}\right. } \end{aligned}$$

We thus obtain, for all $t>0$,

$$\begin{aligned} \frac{\delta }{\delta +\epsilon } [{{\hat{h}}}(0)-{{\hat{g}}}(0)] \le {{\hat{h}}}(t)-{{\hat{g}}}(t)\le \frac{\delta +\epsilon }{\delta }[{{\hat{h}}}(0)-{{\hat{g}}}(0)]. \end{aligned}$$

The monotonicity of ${{\hat{g}}}(t)$ and ${{\hat{h}}}(t)$ then implies

$$\begin{aligned} {{\hat{h}}}(t)\le \frac{\delta +\epsilon }{\delta }[{{\hat{h}}}(0)-{{\hat{g}}}(0)]+{{\hat{g}}}(t)\le {{\hat{h}}}(0)+\frac{\epsilon }{\delta }[{{\hat{h}}}(0)-{{\hat{g}}}(0)]. \end{aligned}$$

Similarly

$$\begin{aligned} {{\hat{g}}}(t)\ge {{\hat{g}}}(0)-\frac{\epsilon }{\delta }[{{\hat{h}}}(0)-{{\hat{g}}}(0)]. \end{aligned}$$

By Step 1 we have

$$\begin{aligned} {{\hat{h}}}(0)-{{\hat{g}}}(0)=h(T)-g(T)+2\epsilon \le 2(M+\epsilon ). \end{aligned}$$

We thus obtain, for all $t>0$,

$$\begin{aligned}{\left\{ \begin{array}{ll} {{\hat{h}}}(t)\le h(T)+\epsilon +\frac{2\epsilon }{\delta }(M+\epsilon ),\\ {{\hat{g}}}(t)\ge g(T)-\epsilon -\frac{2\epsilon }{\delta }(M+\epsilon ). \end{array}\right. } \end{aligned}$$

Since $\theta > {{\hat{u}}}(t,x)\ge \delta$ for all $t>0$ and $x\in [{{\hat{g}}}(t), {{\hat{h}}}(t)]$, it is easily checked that $({{\hat{u}}}(t,x), {{\hat{g}}}(t),{{\hat{h}}}(t))$ and $(u(T+t,x), g(T+t),h(T+t))$ form a pair of upper and lower solutions as described in Lemma 5.2. It follows that

$$\begin{aligned} {[}g(T+t), h(T+t)]\subset ({{\hat{g}}}(t), {{\hat{h}}}(t)) \text{ for } t>0. \end{aligned}$$

Therefore we have, for all $t>T$,

5.35 $$\begin{aligned} {\left\{ \begin{array}{ll} h(t)\le h(T)+\epsilon +\frac{2\epsilon }{\delta }(M+\epsilon ),\\ g(t)\ge g(T)-\epsilon -\frac{2\epsilon }{\delta }(M+\epsilon ). \end{array}\right. } \end{aligned}$$

This implies $h_\infty :=\lim _{t\rightarrow \infty } h(t)$ exists, for otherwise, there exists $t_n\rightarrow \infty$ such that

$$\begin{aligned} h_*:=\liminf _{t\rightarrow \infty } h(t)=\lim _{n\rightarrow \infty } h(t_n)<h^*:=\limsup _{t\rightarrow \infty } h(t). \end{aligned}$$

For any small $\epsilon >0$ we may take $T=t_n$ with *n* sufficiently large such that $h(T)\le h_*+\epsilon$ and (5.35) holds. Then for all $t>T$ we have

$$\begin{aligned} h(t)\le h(T)+\epsilon +\frac{2\epsilon }{\delta }(M+\epsilon )\le h_*+2\epsilon +\frac{2\epsilon }{\delta }(M+\epsilon ). \end{aligned}$$

It follows that $h^*\le h_*+2\epsilon +\frac{2\epsilon }{\delta }(M+\epsilon )$, which is impossible when $\epsilon$ is small enough. We can similarly prove that $g_\infty :=\lim _{t\rightarrow \infty } g(t)$ exists.

For the function $\displaystyle U(t) := \int _{g(t)}^{h(t)} u(t, x) \, dx$, similar to ${{\hat{U}}}(t)$ we have $U(t)=U(0)$ for $t>0$. It follows that

$$\begin{aligned} \int _{-h_0}^{h_0}u_0(x)dx=U(0)=\lim _{t\rightarrow \infty } U(t)=\delta (h_\infty -g_\infty ). \end{aligned}$$

The proof for the case $\delta \in (0, \theta )$ is now complete.

It remains to consider the case $\delta =\theta$. The proof for this case is a simple variation of the arguments in the three steps above. To obtain the corresponding conclusions in Step 1, we simply observe that now the constant triple $(\theta , -h_0-1, h_0+1)$ and (*u*(*t*, *x*), *g*(*t*), *h*(*t*)) form a pair of upper and lower solutions, which yields $u(t,x)\le \theta =\delta$ and $[g(t), h(t)]\subset (-h_0-1, h_0+1)$ for all $t>0$. The corresponding conclusions in Step 2 and Step 3 are proved by the same arguments with considerable simplifications; for example, we can now simply take $({{\hat{u}}}, {{\hat{g}}}, {{\hat{h}}})\equiv (\theta , g(T)-\epsilon , h(T)+\epsilon )$ in Step 3. $\square$

#### 5.6.2 Proof of Theorem 3.5

The proof consists of four steps. The first three steps are based on techniques already used in this paper, but the fourth step relies on a new idea.

**Step 1.** We prove that there exists $M>0$ such that $[g(t), h(t)]\subset (-M, M)$ for all $t>0$ and $\limsup _{t\rightarrow \infty } u(t,x)\le 1$ uniformly for $x\in [g(t), h(t)]$.

We follow the approach in Step 1 of Sect. 3.6.1. Choose ${{\bar{u}}}_0(x)\in X(h_0+1)$ such that

$$\begin{aligned} {{\bar{u}}}_0(x)\ge u_0(x) \text{ for } x\in [-h_0, h_0],\ {{\bar{u}}}_0(x)\ge 1 \text{ for } x\in [-h_0-1, h_0+1]. \end{aligned}$$

Then consider the auxiliary free boundary problem

$$\begin{aligned} {\left\{ \begin{array}{ll} {\tilde{u}}_t-d{\tilde{u}}_{xx}=0, & t>0, \ {\tilde{g}}(t)<x<{\tilde{h}}(t),\\ {\tilde{u}}(t,{\tilde{g}}(t))= {\tilde{u}}(t,{\tilde{h}}(t))=1,& t>0,\\ {\tilde{g}}'(t)=-d {\tilde{u}}_x(t, {\tilde{g}}(t)), & t>0,\\ {\tilde{h}}'(t)=-d {\tilde{u}}_x(t, {\tilde{h}}(t)),& t>0,\\ {\tilde{u}}(0,x)={{\bar{u}}}_0(x),\ -{\tilde{g}}(0)={\tilde{h}}(0)=h_0+1, \end{array}\right. } \end{aligned}$$

By the standard comparison principle we deduce ${\tilde{u}}(t,x)\ge 1$ for $t>0$ and $x\in [{\tilde{g}}(t), {\tilde{h}}(t)]$. Therefore $f(t,x,{\tilde{u}})\le 0$. We may now use Lemma 5.2 to conclude that

5.36 $$\begin{aligned} {[}g(t), h(t)]\subset ({\tilde{g}}(t), {\tilde{h}}(t)),\ u(t,x)\le {\tilde{u}}(t,x) \text{ for } t>0,\ x\in [g(t), h(t)]. \end{aligned}$$

On the other hand, the triple $({\tilde{u}}-1, {\tilde{g}},{\tilde{h}})$ solves a free boundary problem of the form (1.4), and as in Step 1 of Sect. 3.6.1, we can use Du and Lou (2015) to conclude that only vanishing is possible for $({\tilde{u}}-1, {\tilde{g}},{\tilde{h}})$. Hence

$$\begin{aligned}{\left\{ \begin{array}{ll} \lim _{t\rightarrow \infty }[{\tilde{g}}(t), {\tilde{h}}(t)]=[{\tilde{g}}_\infty , {\tilde{h}}_\infty ] \text{ is } \text{ a } \text{ finite } \text{ interval, } \\ {\tilde{u}}(t,x)-1\rightarrow 0 \text{ as } t\rightarrow \infty \text{ uniformly } \text{ for } x\in [{\tilde{g}}(t),{\tilde{h}}(t)]. \end{array}\right. } \end{aligned}$$

The desired conclusions clearly follow from this and (5.36).

**Step 2.** We show that $u(t,x)\rightarrow 1$ as $t\rightarrow \infty$ uniformly for $x\in [g(t), h(t)]$.

Choose ${\hat{f}}(u)\le {\underline{f}}(u)$ such that ${{\hat{f}}}$ satisfies $\mathbf{(f_b)}$. Then, by Lemma 2.1, the following problem

$$\begin{aligned} \left\{ \begin{aligned}&dq'' - cq' + {\hat{f}}(q) = 0, \quad z \in {\mathbb {R}}, \\&q(-\infty ) = 0, \quad q(\infty ) = 1 \end{aligned} \right. \end{aligned}$$

has a solution pair $(c,q)=(c_0,q_0)$ with $c_0>0$ and $q_0(\cdot )$ strictly increasing.

Next, we make use of $q_0(z)$ to construct a lower solution to bound *u*(*t*, *x*) from below as in the proof of Theorem 1.1. Since $q_0(-\infty ) = 0$, we can choose $L > 0$ sufficiently large such that $q_0(h_0 - L) \le \min _{x \in [-h_0, h_0]} u_0(x)$. Define

$$\begin{aligned} {\underline{u}}(t, x):= \max \{q_0(ct - x - L), q_0(ct + x - L)\}. \end{aligned}$$

Due to ${\hat{f}} (u)\le f(t,x,u)$ we see that ${\underline{u}}(t, x)$ satisfies (in the weak sense)

$$\begin{aligned} {\underline{u}}_t \le d {\underline{u}}_{xx} + f(t,x, {\underline{u}}) \quad \text {for } t > 0, \; x \in {\mathbb {R}}. \end{aligned}$$

Moreover

$$\begin{aligned} 0< {\underline{u}}(t, x)<1 \quad \text {for } x \in {\mathbb {R}},\ \text{ which } \text{ implies } {\underline{u}}(t, x)< u(t,x) \text{ for } t>0,\ x\in \{g(t), h(t)\},\end{aligned}$$

and

$$\begin{aligned} {\underline{u}}(0, x) = \max \{q_0(-x - L), q_0(x - L)\} \le q_0(h_0 - L) \le u_0(x) \quad \text {for } x \in [-h_0, h_0]. \end{aligned}$$

Therefore we can apply the standard comparison principle over $\{(t, x): t > 0, x \in [g(t), h(t)]\}$ to deduce $u(t, x) \ge {\underline{u}}(t, x)$ in this region. Since

$$\begin{aligned} \lim _{t \rightarrow \infty } \Vert {\underline{u}}(t,\cdot ) - 1\Vert _{L^\infty ({\mathbb {R}})} = 0, \end{aligned}$$

we obtain

$$\begin{aligned} \liminf _{t\rightarrow \infty }{u}(t, x) \ge \lim _{t\rightarrow \infty }{\underline{u}}(t, x) =1 \quad \text{ uniformly } \text{ for } x \in [g(t), h(t)]. \end{aligned}$$

The desired conclusion now follows from this and Step 1.

**Step 3.** We show that, as $t \rightarrow \infty$, $[g(t), h(t)] \rightarrow [g_\infty , h_\infty ] \text{ is } \text{ a } \text{ finite } \text{ interval. }$

We follow Step 3 of Sect. 3.6.1. For any small $\epsilon >0$, we can find $T=T_\epsilon >0$ large such that

$$\begin{aligned} u(t,x)\in [1-\epsilon , 1+\epsilon ] \text{ for } t\ge T, \ x\in [g(t), h(t)]. \end{aligned}$$

Next we consider the following auxiliary problem

5.37 $$\begin{aligned} {\left\{ \begin{array}{ll} {{\hat{u}}}_t-d{{\hat{u}}}_{xx}=0, & t>0, \ {{\hat{g}}}(t)<x<{{\hat{h}}}(t),\\ {{\hat{u}}}(t,{{\hat{g}}}(t))= {{\hat{u}}}(t,{{\hat{h}}}(t))=1,& t>0,\\ {{\hat{g}}}'(t)=-d{{\hat{u}}}_x(t, {{\hat{g}}}(t)), & t>0,\\ {\hat{h}}'(t)=-d {{\hat{u}}}_x(t, {{\hat{h}}}(t)),& t>0,\\ {\hat{u}}(0,x)={{\hat{u}}}_0(x),\ {{\hat{g}}}(0)=g(T)-\epsilon , {{\hat{h}}}(0)=h(T)+\epsilon ,& {{\hat{g}}}(0)\le x\le {{\hat{h}}}(0), \end{array}\right. } \end{aligned}$$

where ${{\hat{u}}}_0$ is a $C^2$ function satisfying

$$\begin{aligned} {{\hat{u}}}_0({{\hat{g}}}(0))={{\hat{u}}}_0({{\hat{h}}}(0))=1,\ \max \{1, u(T, x)\} \le {{\hat{u}}}_0(x)\le 1+\epsilon \text{ for } x\in [{{\hat{g}}}(0), {{\hat{h}}}(0)]. \end{aligned}$$

We easily see that the unique solution $({{\hat{u}}}, {{\hat{g}}}, {{\hat{h}}})$ of (5.37) satisfies $1+\epsilon \ge {{\hat{u}}}(t,x)\ge 1$ for all $t>0$ and $x\in [{{\hat{g}}}(t), {{\hat{h}}}(t)]$. It follows that ${{\hat{h}}}'(t)\ge 0\ge {{\hat{g}}}'(t)$ for $t>0$. As in Step 3 of Sect. 3.6.1, the function $\displaystyle {{\hat{U}}}(t):=\int _{{{\hat{g}}}(t)}^{{{\hat{h}}}(t)} {{\hat{u}}}(t,x)dx$ satisfies ${{\hat{U}}}(t)={{\hat{U}}}(0)$ for $t>0$, which leads to, for all $t>0$,

$$\begin{aligned}{\left\{ \begin{array}{ll} {{\hat{h}}}(t)\le h(T)+\epsilon +2\epsilon (M+\epsilon ),\\ {{\hat{g}}}(t)\ge g(T)-\epsilon -2\epsilon (M+\epsilon ). \end{array}\right. } \end{aligned}$$

Since ${{\hat{u}}}(t,x)\ge 1$ for all $t>0$ and $x\in [{{\hat{g}}}(t), {{\hat{h}}}(t)]$, it is easily checked that $({{\hat{u}}}(t,x), {{\hat{g}}}(t),{{\hat{h}}}(t))$ and $(u(T+t,x), g(T+t),h(T+t))$ form a pair of upper and lower solutions as described in Lemma 5.2. It follows that

$$\begin{aligned} {[}g(T+t), h(T+t)]\subset ({{\hat{g}}}(t), {{\hat{h}}}(t)) \text{ for } t>0. \end{aligned}$$

Therefore we have, for all $t>T$,

$$\begin{aligned}{\left\{ \begin{array}{ll} h(t)\le h(T)+\epsilon +2\epsilon (M+\epsilon ),\\ g(t)\ge g(T)-\epsilon -2\epsilon (M+\epsilon ). \end{array}\right. } \end{aligned}$$

This implies, as in Step 3 of Sect. 3.6.1, that $h_\infty :=\lim _{t\rightarrow \infty } h(t)$ and $g_\infty :=\lim _{t\rightarrow \infty } g(t)$ both exist.

**Step 4.** We prove $h_\infty -g_\infty >0$.

As before, the function $\displaystyle U(t) := \int _{g(t)}^{h(t)} u(t, x) \, dx$ satisfies, for $t >0$,

$$\begin{aligned}\begin{aligned} U'(t)&=\int _{g(t)}^{h(t)} f(t,x,u(t,x)) \, dx \ge \int _{g(t)}^{h(t)} {\underline{f}}(u(t,x)) dx. \end{aligned} \end{aligned}$$

If $u_0(x)\ge 1$ for $x\in [-h_0, h_0]$, then by a simple comparison consideration we deduce $u(t,x)\ge 1$ for $t>0$ and $x\in [g(t), h(t)]$, which implies $h'(t)\ge 0\ge g'(t)$ for $t>0$. Therefore $h_\infty \ge h(0)>g(0)\ge g_\infty$.

If $u_0(x)\le 1$ for $x\in [-h_0, h_0]$, then by a simple comparison consideration we deduce $u(t,x)\le 1$ for $t>0$ and $x\in [g(t), h(t)]$. Since ${\underline{f}}$ satisfies $\mathbf{(f_A)}$, we have ${\underline{f}}(u)>0$ for $u\in ({\underline{\theta }}, 1)$ with some fixed ${\underline{\theta }}\in [0, 1)$. By the conclusion of Step 2, we can find $T_0\ge 0$ such that $1\ge u(t,x)>{\underline{\theta }}$ for $t\ge T_0$ and $x\in [g(t), h(t)]$. It follows that

$$\begin{aligned} U'(t)\ge \int _{g(t)}^{h(t)} {\underline{f}}(u(t,x)) dx\ge 0 \text{ for } t\ge T_0, \end{aligned}$$

with $U'(t)\equiv 0$ for $t\ge T_0$ only if $u(t,x)\equiv 1$ for such *t*. In the case $u(t,x)\equiv 1$ for $t\ge T_0$, it follows that $h(t)\equiv h(T_0)=h_\infty >g_\infty =g(T_0)\equiv g(t)$ for $t>T_0$, and otherwise we have $U(\infty )>U(T_0)>0$ which implies $h_\infty >g_\infty$.

If $u_0(x)-1$ changes sign in $[-h_0, h_0]$, then a new idea is required. Now $m_0:=\max _{x\in [-h_0, h_0]}u_0(x)>1$. Let *v*(*t*) be the unique solution of

$$\begin{aligned} v'={{\bar{f}}}(v),\ v(0)=m_0. \end{aligned}$$

Then from ${{\bar{f}}}(1)=0$ and ${{\bar{f}}}(u)<0$ for $u>1$ we see that *v*(*t*) decreases to 1 as $t\rightarrow \infty$. Moreover, the usual comparison principle gives $u(t,x)\le v(t)$ for $t>0$ and $x\in [g(t), h(t)]$.

Since ${\underline{f}}'(1)<0$, there exists $\epsilon _1\in (0,\varepsilon ]$ (where $\varepsilon$ is given in (3.5)) such that ${\underline{f}}(u)$ is decreasing in $[1-\epsilon _1, 1+\epsilon _1]$. Using $u, v\rightarrow 1$ as $t\rightarrow \infty$, we can find $T>0$ large so that *u*(*t*, *x*), *v*(*t*) both belong to $[1-\epsilon _1, 1+\epsilon _1]$ for $t\ge T$. Therefore, by the extra assumption (3.5), for $t\ge T$,

$$\begin{aligned} \begin{aligned} U'(t)&\ge \int _{g(t)}^{h(t)} {\underline{f}}(u(t,x)) dx\ge \int _{g(t)}^{h(t)} {\underline{f}}(v(t)) dx\\&\ge \varepsilon ^{-1} [h(t)-g(t)]{{\bar{f}}}(v(t))=\varepsilon ^{-1} [h(t)-g(t)]v'(t), \end{aligned} \end{aligned}$$

and so, for any $t>s\ge T$,

$$\begin{aligned} \int _{g(t)}^{h(t)}u(t,x)dx-\int _{g(s)}^{h(s)}u(s,x)dx=U(t)-U(s)\ge \varepsilon ^{-1}\int _s^t[h(\tau )-g(\tau )]v'(\tau )d\tau . \end{aligned}$$

Assuming $h_\infty =g_\infty$, we now deduce a contradiction. Using $0<h(t)-g(t)\rightarrow 0$ as $t\rightarrow \infty$, we can find a positive sequence $t_n\rightarrow \infty$ such that $h(t_n)-g(t_n)\ge h(t)-g(t)$ for $t\ge t_n$. Therefore for all large *n* and $t>t_n$, in view of $v'<0$, we have

$$\begin{aligned} \int _{t_n}^t[h(\tau )-g(\tau )]v'(\tau )d\tau \ge [h(t_n)-g(t_n)]\int _{t_n}^tv'(\tau )d\tau \ge [h(t_n)-g(t_n)](1-v(t_n)). \end{aligned}$$

It follows that, for all large *n* and $t>t_n$,

5.38 $$\begin{aligned} \begin{aligned} \int _{g(t)}^{h(t)}u(t,x)dx&\ge \int _{g(t_n)}^{h(t_n)}u(t_n,x)dx+\varepsilon ^{-1}[h(t_n)-g(t_n)](1-v(t_n))\\&=\int _{g(t_n)}^{h(t_n)}[u(t_n,x)+\varepsilon ^{-1}(1-v(t_n))]dx. \end{aligned} \end{aligned}$$

Since $u(t,x), v(t)\rightarrow 1$ as $t\rightarrow \infty$ uniformly in $x\in [g(t), h(t)]$, we can fix *n* sufficiently large such that

$$\begin{aligned} u(t_n,x)+\varepsilon ^{-1}(1-v(t_n))>0 \text{ for } x\in [g(t_n), h(t_n)],\end{aligned}$$

which implies

$$\begin{aligned} I_n:=\int _{g(t_n)}^{h(t_n)}[u(t_n,x)dx+\varepsilon ^{-1}(1-v(t_n))]dx>0. \end{aligned}$$

On the other hand, $h_\infty =g_\infty$ and $u(t,x)\rightarrow 1$ as $t\rightarrow \infty$ imply $\displaystyle \int _{g(t)}^{h(t)}u(t,x)dx\rightarrow 0$ as $t\rightarrow \infty$. Thus letting $t\rightarrow \infty$ in (5.38) we deduce $0\ge I_n>0$. This contradiction proves $h_\infty >g_\infty$. The proof is now complete. $\square$

## Footnotes

1 There does not appear to be a widely agreed definition for super invaders. In this paper, we use this term for invaders which can successfully spread into new territories without being significantly affected by the Allee effects, or by variations of the environment, or by small initial population size.

2 In the simulations here, we use standard finite difference method for the reaction–diffusion equation, combined with the front tracking method for the moving boundary. Such a combination has been developed and successfully used in Liu and Liu (2018), Liu et al. (2020), Khan et al. (2021) for related models. One could also combine finite difference with the front fixing method of Piqueras et al. (2017). Some comparisons of these two approaches can be found in Liu and Liu (2018).

3 This contrasts interestingly to the vanishing case for (1.4), where as $t\rightarrow \infty$, $u(t,x)\rightarrow 0$ uniformly and [*g*(*t*), *h*(*t*)] converges to a finite interval $[g_\infty , h_\infty ]\supset [-h_0, h_0]$.
